# Discriminant validity, responsiveness and reliability of the arthritis-specific Work Productivity Survey assessing workplace and household productivity within and outside the home in patients with axial spondyloarthritis, including nonradiographic axial spondyloarthritis and ankylosing spondylitis

**DOI:** 10.1186/ar4680

**Published:** 2014-08-06

**Authors:** Jane T Osterhaus, Oana Purcaru

**Affiliations:** Wasatch Health Outcomes, 2613 Silver Cloud Drive, Park City, UT 84060 USA; UCB Pharma, Allee de la Recherche, 60, 1070 Brussels, Belgium

## Abstract

**Introduction:**

The arthritis-specific Work Productivity Survey (WPS) was developed to evaluate productivity limitations associated with arthritis within and outside the home. There is an unmet need for an instrument assessing similar productivity limitations in axial spondyloarthritis (axSpA), including nonradiographic axSpA and ankylosing spondylitis. Following its validation in rheumatoid and psoriatic arthritis, we aimed to assess psychometric properties of WPS in adult-onset active axSpA in this analysis.

**Methods:**

Psychometric properties were assessed using data from the RAPID-axSpA trial (NCT01087762) in which researchers investigated certolizumab pegol efficacy and safety in axSpA. WPS was completed at baseline and every 4 weeks until week 24. Validity was evaluated at study baseline via known-groups defined by the first and third quartile cutoffs of patient scores to Ankylosing Spondylitis Disease Activity Score (ASDAS), Bath Ankylosing Spondylitis Disease Activity Index (BASDAI), back pain, Bath Ankylosing Spondylitis Functional Index (BASFI), Short Form 36 health survey (SF-36) and Ankylosing Spondylitis Quality of Life Scale (ASQoL). Responsiveness and reliability were assessed by comparing WPS mean changes in ASAS 20% improvement criteria (ASAS20), BASDAI50, ASDAS clinically important improvement/major improvement (CII/MI) and BASFI minimum clinically important difference (MCID) responders versus nonresponders at week 12. All comparisons were conducted on observed cases in the randomized set using a nonparametric bootstrap-t method.

**Results:**

The results confirmed the psychometric properties of WPS. AxSpA patients with a worse health state had significantly more days of household work lost, household work with reduced productivity, social activities missed and outside help hired, as well as a higher interference rate of arthritis, than patients with a better health state. Similarly, employed patients with a worse health state had significantly more work days lost or with productivity reduced, and a higher interference of arthritis on work productivity. Similar findings were also observed in the nonradiographic (nr) axSpA and AS subpopulations. The WPS was responsive to clinical changes, with responders reporting larger improvements at week 12 in WPS scores versus nonresponders. Effect sizes in responders were generally moderate to large (standardized response mean >0.5).

**Conclusions:**

These analyses demonstrate that WPS is a valid, responsive and reliable instrument for the measurement of productivity within and outside the home in adult-onset axSpA, as well as the in subpopulations of AS and nr-axSpA.

**Electronic supplementary material:**

The online version of this article (doi:10.1186/ar4680) contains supplementary material, which is available to authorized users.

## Introduction

*Axial spondyloarthritis* (axSpA) refers to spondyloarthritis with predominantly axial involvement and comprises the well-known disease subgroup *ankylosing spondylitis* (AS), as well as a disease subgroup with little or no changes on plain radiographs, referred to as *nonradiographic axial spondyloarthritis* (nr-axSpA). Nr-axSpA and AS can be considered opposite ends of the same disease spectrum [1]. According to this concept, the presence of radiographic changes in the sacroiliac joints (and the presence of syndesmophytes in the spine) should be regarded as markers of disease progression and severity rather than as essential diagnostic criteria.

AS, the most frequently investigated subset of axSpA, is a chronic inflammatory rheumatic disease that affects approximately twice as many men as women and has a disease onset usually in the second and third decades of life. The prevalence of AS worldwide ranges from 0.1% to 1.4% [2–4]. The prevalence of spondyloarthritis in the United States was recently shown to be 1.4% [5]. Disability in AS is related to the degree of inflammatory activity causing pain, stiffness, fatigue and poor quality of sleep, as well as to the degree of bony ankylosis causing loss of spinal mobility. During early disease stages, disability is determined mostly by inflammatory activity, whereas in long-standing disease, both inflammation and ankylosis contribute to disability. The average time between onset of symptoms and definite disease diagnosis of AS has been reported to be up to 9 years [6]. At least 30% of patients have severe disease which is often associated with considerable loss of function.

Two of the common symptoms associated with AS—pain and fatigue—are expected to impact work-related performance. Fatigue in patients with AS has been reported to be associated with limitations in daily life, functioning, pain and stiffness, as well as with global well-being and mental health [7]. AS patients in one study ranked “impact on work” as the area of their life most affected by their condition [8].

It has been reported that the costs associated with work disability or productivity losses at paid work (indirect costs) of AS are higher in some countries than the direct medical costs [9]. In a recently reported study conducted in the Netherlands of patients with AS under the care of rheumatologists, 11.6% of patients with paid work had an episode of AS-related sick leave in the previous 2 weeks (absenteeism) and just over 50% felt their work was adversely influenced by AS, suggesting a significant impact on presenteeism [10]. In the entire sample, 71% experienced restrictions in different types of unpaid tasks. Limitations in physical function were consistently associated with work outcome.

The key goals of treatment in AS include control of pain and stiffness, as well as reducing damage, disability and loss of function. Given that AS tends to occur in the second and third decades of life, it is expected that many people initially diagnosed with AS are in the midst of their working careers. In a 2001 review, Boonen *et al.* summarized findings on work participation among AS patients in different countries [11]. The proportion of patients in employment ranged from 34% to 96%, and the proportion of patients with work disability ranged from 3% to 50%.

In order to fully quantify the impact of an intervention on productivity, it is crucial to consider the entire productivity continuum, both within the work environment and within the home [12]. Preventing disability and loss of function may improve a patient’s ability to stay in the workforce or maintain the ability to live independently at home. Therefore, there is interest in understanding the impact of axSpA and potential axSpA treatments on work-related productivity, including paid work as well as household work.

Historically, there has been an unmet need for an instrument designed to assess presenteeism and absenteeism in both the work and home environments [13–16]. The arthritis-specific Work Productivity Survey (WPS) was developed to fulfill the unmet need for an arthritis-specific instrument to assess the impact of an intervention on productivity within the work and home environments, in addition to daily activities during the preceding month [17]. Details of the development of the WPS are reported elsewhere [17]. The WPS has demonstrated properties of discriminative validity, reliability and responsiveness for the measurement of productivity within and outside the home in patients with active rheumatoid arthritis (RA) and psoriatic arthritis (PsA) [17–19].

There is no gold standard measure for assessing productivity in axSpA. During the Outcome Measures in Rheumatology (OMERACT) 9 meeting, the WPS was one of six instruments identified by the OMERACT Worker Productivity group as a possible candidate for assessing worker productivity changes in rheumatology, based on the available filter evidence (truth, discrimination and feasibility) [20].

The WPS was selected to measure the impact of axSpA on workplace and household productivity, as well as on participation in daily activities, because of the ease of use and positive response in terms of psychometric properties seen in rheumatoid arthritis (RA) [17], and the similarity in terms of work disability associated with RA and axSpA.

Our objective in writing this article was to assess the discriminant validity, responsiveness and reliability of the Work Productivity Survey in adult-onset active axSpA, as well as in AS and nr-axSpA subpopulations.

## Methods

### Patients and study design

Data from the double-blind period of the RAPID-axSpA (efficacy and safety of certolizumab pegol (CZP) in axSpA) trial (double-blind and placebo-controlled to week 24, dose-blind to week 48 and then open-label to week 204) were used to conduct the psychometric validation of WPS [21]. In the first 24 weeks of RAPID-axSpA, CZP 200 mg every 2 weeks (Q2W), 400 mg every 4 weeks (Q4W) or placebo were investigated. The trial was conducted at 83 centers across North America, Latin America, Western Europe and Central/Eastern Europe from March 2010 to October 2011. Institutional review boards or ethics committees approved the protocol at each center (see Additional file 1). All patients gave written consent, and the study was conducted in accordance with the Declaration of Helsinki [21].

Patients were randomly assigned 1:1:1 to receive (1) placebo (saline) Q2W, or subcutaneous CZP 400 mg at Weeks 0, 2 and 4 (loading dose) followed by either (2) 200 mg CZP Q2W or (3) 400 mg CZP Q4W until Week 24. Placebo patients who did not achieve at least Assessment of SpondyloArthritis International Society (ASAS) 20% improvement criteria (ASAS20) response at both week 14 and week 16 remained blinded, but were rerandomized to active treatment at week 16 in a 1:1 ratio (CZP 200 mg Q2W or CZP 400 mg Q4W) and received the loading dose at weeks 16, 18 and 20. CZP patients continued to receive the initially assigned dose [21].

The primary efficacy endpoint was the ASAS20 response at week 12 [22, 23]. Secondary and exploratory endpoints included the ASAS40, ASAS50, ASAS70, Total and Nocturnal Spinal Pain, physical functioning assessed by Bath Ankylosing Spondylitis Functional Index (BASFI), Bath Ankylosing Spondylitis Disease Activity Index (BASDAI) and Ankylosing Spondylitis Disease Activity Score (ASDAS), health-related quality of life (HRQoL), assessed using the Short Form 36-item health survey (SF-36), EuroQoL 5 dimensions (EQ-5D) and the Ankylosing Spondylitis Quality of Life Scale (ASQoL), and productivity measured using the WPS.

### Questionnaires

The WPS is a disease-specific questionnaire used to assess the impact of arthritis on workplace and household productivity, as well as daily activities during the preceding month. It is interviewer-administered and self-reported by the patient and has a 1-month recall period [17].

The first item of the WPS addresses current labor market participation (employment outside the home), as well as providing normative and comparative data on employment status. This is a strong indicator of ability to work, because not working implies complete loss of paid productivity. Two items capture self-reported absenteeism (days of work missed) and presenteeism (days with productivity reduced by at least half) due to arthritis, and two items capture the same concepts but apply to nonpaid (household) work. Additional items capture the respondent’s estimate of the extent to which arthritis has interfered with work productivity (paid and nonpaid) on a scale of 0 to 10 (0 = no interference and 10 = complete interference), the number of days in the past month that outside help was hired because of arthritis, and the number of days in the past month family, social or leisure activities were missed because of arthritis [17].

The ASDAS is a composite score derived from a number of assessments, which are scored by the subject and physician and multiplied by a proven formula, with lower scores indicating low disease activity [24]. The BASFI comprises 10 items assessing physical function over the preceding week [25]. The summary score from this scale is the mean of the 10 items and ranges from 0 to 10, with 0 representing the best state (lower disease activity) and 10 the worst state. The minimum clinically important difference (MCID) for BASFI is one point.

The BASDAI is the most commonly used instrument to measure the disease activity of ankylosing spondylitis over the preceding week, and ranges from 0 to 10, with 0 representing the best state and 10 the worst state. The BASDAI50 is defined as an improvement of at least 50% in the BASDAI compared to baseline. A response criterion for the BASDAI is defined by an MCID decrease of at least one [26].

Total and Nocturnal Spinal Pain are assessed by two questions rated on a 0 to 10 numerical rating scale (NRS), where 0 = no pain and 10 = most severe pain. The ASQoL is an 18-item questionnaire, each item of which is used to assess the patient’s current opinion on his or her quality of life [27]. Each item is scored as 1 = yes or 0 = no. The summary score is the total of the yes and no scores, thus ranging from 0 (best HRQoL) to 18 (worst HRQoL).

The SF-36 is a widely used generic HRQoL instrument used to evaluate eight health domains: physical functioning, role physical, bodily pain, general health, vitality, social functioning, role emotional and mental health [28]. The eight domains are summarized in two component summaries: the Physical Component Summary (PCS) and the Mental Component Summary (MCS) [26]. Scores on the SF-36 range between 0 and 100, with higher scores indicating a better HRQoL.

The EQ-5D questionnaire is comprised of a five-item health status measure and a Visual Analogue Scale (VAS). Each of the five dimensions is divided into three levels: no problem, some or moderate problems and extreme problems, scored as 1, 2, and 3, respectively. The EQ-5D VAS records the respondent’s self-rated health status on a vertical 20-cm scale, 0 to 100 graduated (0 = worst imaginable health status and 100 = best imaginable health status).

The ASAS20 response is defined as an improvement of at least 20% and absolute improvement of at least one unit on a 0 to 10 NRS in at least three of four domains: patient’s global assessment of disease Activity, total spinal pain NRS score, BASFI and mean of BASDAI questions 5 and 6 concerning morning stiffness intensity and duration and the absence of deterioration in the potential remaining domain, with deterioration defined as a relative worsening of at least 20% and an absolute worsening of at least one unit [29].

The ASAS criteria for 40%, 50% or 70% improvement are defined as relative improvements of at least 40%, 50% or 70% and absolute improvement of at least two units on a 0 to 10 NRS in at least three of the four domains and no worsening at all in the remaining domain.

Further details of the questionnaires assessed are included in Additional file 2.

### Data handling and statistical analysis

The assessment of the psychometric properties (discriminant validity, responsiveness and reliability) of the WPS was performed on the overall axSpA randomized set (RS) population, regardless of the randomization group. Analyses were also performed on the nr-axSpA and AS subpopulations separately.

### Discriminant validity

Given the nature of the WPS questionnaire, which is composed of several single-global questions, scored and interpreted separately, and the length of the recall period of these questions, the construct validity of the WPS questionnaire was evaluated by means of discriminant validity using correlations and the known-groups validation method. The association between the responses to the WPS questions (Q)2 to Q9 and scores of the different measures of disease activity, physical functioning or HRQoL was assessed using Kendall correlation coefficients. Given the difference between the concepts assessed by the WPS questions and the other measures considered, the correlation coefficients are expected *a priori* to be low to moderate (low = 0 to 0.3, moderate ≥0.3 to <0.5), thus indicating a divergent validity of the measures compared. High correlations could imply a low discriminant validity and suggest that two items are measuring similar concepts. The Kendall association coefficients were evaluated between WPS Q2 to Q9 and the following selected measures: ASDAS, BASDAI, BASFI, total/nocturnal spine pain, SF-36 MCS, PCS and domains, ASQoL, fatigue NRS (from BASDAI) and EQ-5D VAS.

The known-groups validity method was used to compare the productivity scores between patients with a worse health state versus patients with a better health state. A patient with a worse health state was considered to be a patient with a higher disease activity, a worse HRQoL level or a lower physical functioning level, whereas a patient with a better health state was defined as having either a lower disease activity, a better HRQoL or a higher physical functioning level, respectively. The assumption tested through the known-groups validity method was that patients with a worse health state were expected *a priori* to have higher losses in paid and household work productivity (that is, higher WPS scores to Q2 to Q9) due to their disease, compared with patients in a better health state. For this purpose, known groups were formed using the first- and third-quartile scores for each outcome as cutoff points in order to avoid comparison of unbalanced groups [17]. Patients with baseline SF-36 scores at or above the third quartile, or ASQoL or fatigue NRS (from BASDAI) scores at or below the first quartile, were considered to have a “better” HRQoL, and those with SF-36 scores at or below the first quartile or ASQoL or fatigue NRS scores at or above the third quartile were defined as having “worse” HRQoL. Similarly, “better” and “worse” physical function were defined as BASFI scores at or below the first quartile and at or above the third quartile, or SF-36 physical function or SF-36 PCS scores at or above the third quartile and at or below the first quartile, respectively. Patients with ASDAS, BASDAI, or total/nocturnal spine pain score at or below the first quartile were considered to have “low” disease activity/severity, whereas ASDAS, BASDAI, or total/nocturnal spine pain score at or above the third quartile indicated “high” disease activity/severity.

The discriminant validity of the WPS was assessed using baseline observed data. To test the validity of productivity at paid work (WPS Q2-Q4), cutoff points were computed only on the patients employed outside the home, whereas the thresholds were computed on all patients for productivity within the home (Q5-Q9). Sensitivity analyses were performed using a median cutoff threshold.

A nonparametric bootstrap-t method was used to compare the mean WPS question responses between the known groups [30]. This method was favored because of the highly skewed distribution of the WPS scores. Bootstrap analyses were performed with 10,000 replications. A variance stabilizing transformation was used in order to adjust for dependence between the bootstrap values and the corresponding standard error.

### Responsiveness to clinical changes and reliability

The responsiveness of the WPS to clinical changes in a patient’s condition over time was evaluated by comparing the changes from baseline in productivity scores between clinical responders versus nonresponders at week 12 (as measured by ASAS20 criteria). The assumption tested was that clinical responders would have higher improvements in productivity at work outside the home and within the household versus nonresponders, reflected by higher negative changes (in absolute value) in WPS scores.

According to the primary analysis, patients were considered a “responder” if they met the criteria of ASAS20 improvement from baseline at week 12. Any patient who did not meet the criteria for ASAS20 was considered a “nonresponder”.

The reliability of the WPS was tested in conjunction with the responsiveness to the ASAS20 clinical response by comparing the changes in WPS scores in patients achieving ASAS40, ASAS50, BASDAI50, ASDAS major improvement (MI), ASDAS clinically important improvement (CII), total/nocturnal back pain MCID and BASFI MCID responses at week 12 versus nonresponders.

WPS score changes from baseline at week 12 were compared between week 12 clinical responders versus nonresponders using a nonparametric bootstrap-t method. A variance stabilizing transformation was used in order to adjust for dependence observed between bootstrap values and the corresponding standard error. Bootstrap analyses were performed with 10,000 replications.

In addition to the comparison of the changes in WPS scores between the clinical responders and nonresponders, the standardized response mean (SRM) was calculated. The SRM is one of the most widely used measures of the effect size of the response, indicating whether the change was large relative to the variability of the measurements. The SRM is estimated as the mean change in scores between two visits divided by the standard deviation of that change in scores. Thresholds for the SRM (absolute values) were proposed by Cohen [31] to interpret the size of the effects: “small” from 0.2 to 0.5, “moderate” from 0.5 to 0.8 and “large” greater than 0.8.

The responsiveness and reliability of the WPS was assessed at week 12 on all RS patients, regardless of the randomization group.

## Results

### Patient characteristics

A total of 325 patients were randomized, and 298 (91.7%) patients completed the 24-week phase. In the overall axSpA population, RS patients had a mean age of approximately 39.6 years, with 78.8% of patients between ages 25 and 54 years. Over half (61.5%) of the patients were male, and most (90.2%) were white (Table 1). In the AS subpopulation, the mean age (41.5 years) was higher compared to the nr-axSpA subpopulation (37.4 years), and AS patients were also more likely to be male compared to nr-axSpA patients (72.5% versus 48.3%, respectively) (Table 1).

**Table 1:** **Baseline demographics and clinical characteristics in axial spondyloarthritis**, **ankylosing spondylitis and nonradiographic axial spondyloarthritis populations (randomized sets, observed cases)** ^**a**^

	axSpA (***n*** = 325)	AS (***n*** = 178)	nr-axSpA (***n*** = 147)
Demographic characteristics			
Age, yr	39.6 ± 11.9	41.5 ± 11.6	37.4 ± 11.8
Sex, % male	61.5	72.5	48.3
Race, % white	90.2	89.3	91.2
Disease characteristics			
Time from axSpA diagnosis^b^ (yr), median (min–max)	3.9 (0.0 to 37.9)	5.5 (0.2 to 37.9)	2.5 (0.0 to 32.2)
Symptom duration (yr), median (min–max)	7.7 (0.3 to 50.9)	9.1 (0.3 to 50.9)	5.5 (0.3 to 41.5)
CRP^c^ (mg/L), median (min–max)	13.9 (0.1 to 174.8)	14.3 (0.1 to 174.8)	11.9 (0.1 to 156.2)
Positive for HLA-B27, *n* (%)	255 (78.5)	145 (81.5)	110 (74.8)
BASDAI	6.5 ± 1.6	6.4 ± 1.6	6.5 ± 1.5
BASFI	5.4 ± 2.2	5.7 ± 2.2	4.9 ± 2.3
BASMI	3.8 ± 1.7	4.4 ± 1.7	3.2 ± 1.5
ASDAS	3.8 ± 1.0	3.9 ± 0.9	3.7 ± 1.0
Total spine pain^d^	7.0 ± 1.9	7.1 ± 1.9	7.0 ± 1.9
Nocturnal spine pain^d^	6.9 ± 2.3	6.9 ± 2.2	6.9 ± 2.5
Prior TNF inhibitor exposure, %	16.0	20.2	10.9
Health-related quality of life			
Fatigue NRS^d^	6.7 ± 1.9	6.6 ± 2.0	6.7 ± 1.8
SF-36 MCS^d^	40.5 ± 12.1	40.9 ± 12.0	40.0 ± 12.3
SF-36 PCS^d^	32.5 ± 7.5	32.0 ± 7.3	33.1 ± 7.8
ASQoL^d^	11.8 ± 4.3	11.8 ± 4.3	11.7 ± 4.3
EQ-5D VAS	48.3 ± 19.4	47.3 ± 18.5	49.4 ± 20.4

^a^ASDAS: Ankylosing spondylitis disease activity scale; ASQoL: Ankylosing spondylitis quality of life; axSpA, Axial spondyloarthritis; BASDAI: Bath ankylosing spondylitis disease activity index; BASFI: Bath ankylosing spondylitis functional index; BASMI: Bath ankylosing spondylitis metrology index; CRP: C-reactive protein; EQ-5D: EuroQoL 5 dimensions; HLA: Human leukocyte antigen; MCS: Mental component summary; nr-axSpA, Nonradiographic axial spondyloarthritis; NRS: Numerical rating scale; PCS: Physical component summary; SF-36: Short form 36 item; TNF: Tumor necrosis factor; VAS: Visual analogue scale. ^b^From the start date of the primary disease. ^c^Normal range of CRP <8.0 mg/L. ^d^In full analysis set population: *n* = 324 axSpA, 178 AS, 146 nr-axSpA. Except where indicated otherwise, values are the mean ± SD. There were no significant differences between treatment groups at baseline.

Patients in the overall axSpA population reported a median time since disease diagnosis of 3.9 years. In the AS subpopulation, the median time since diagnosis was 5.5 years, and for the nr-axSpA subpopulation it was 2.5 years (Table 1). The majority (78.5%) of patients in the overall axSpA population tested positive for human leukocyte antigen (HLA) B27; this was also true for the AS and nr-axSpA subpopulations (81.5% and 74.8%, respectively). In general, BASDAI scores were similar, whereas BASMI and BASFI scores were lower, in the nr-axSpA subpopulation relative to the AS subpopulation, indicating comparable disease burden but less limitation in function and mobility in patients with nr-axSpA (Table 1).

Whereas the largest percentage of patients in the overall axSpA population and AS subpopulation were from the Eastern Europe (43.4% and 55.1%, respectively), patients in the nr-axSpA subpopulation were more evenly distributed among North Americans (27.2%), Western Europeans (34.7%), and Eastern Europeans (29.3%).

At baseline, 69.2% of patients in the overall axSpA population were employed outside the home, 12.3% were unable to work due to axSpA, 5.8% were students and 5.5% were retired. The rest were homemakers (3.1%), unable to work due to non-axSpA health problems (1.9%) or had other nonemployment status (2.2%) (Table 2). Generally similar employment rates were noted in the nr-axSpA and AS subpopulation, although in the nr-axSpA subpopulation there were slightly more patients employed outside the home, homemakers or students, as well as fewer patients who were unable to work due to arthritis or were retired compared to the AS subpopulation (Table 2).

**Table 2:** **Employment status at baseline in axial spondyloarthritis**, **ankylosing spondylitis and nonradiographic axial spondyloarthritis populations (randomized sets, observed cases)** ^**a**^

	axSpA (***n*** = 325)	AS (n = 178)	nr-axSpA (n = 147)
Employment status, n (%)			
Employed outside the home	225 (69.2)	120 (67.4)	105 (71.4)
Not employed outside home			
Homemakers	10 (3.1)	4 (2.2)	6 (4.1)
Retired	18 (5.5)	12 (6.7)	6 (4.1)
Student	19 (5.8)	6 (3.4)	13 (8.8)
Unable to work due to arthritis	40 (12.3)	28 (15.7)	12 (8.2)
Unable to work due to non-arthritis-related health problems	6 (1.8)	5 (2.8)	1 (0.7)
Other nonemployed status	7 (2.2)	3 (1.7)	4 (2.7)
Job function if employed, *n* (%)			
Nonmanual	109 (48.4)	53 (44.2)	56 (53.3)
Mixed (manual and nonmanual)	73 (32.4)	40 (33.3)	33 (31.4)
Manual with no supervisory duties	43 (19.1)	27 (22.5)	16 (15.2)

^a^AS, Ankylosing spondylitis; axSpA, Axial spondyloarthritis, nr-axSpA, Nonradiographic axial spondyloarthritis. Employment status was captured through the Work Productivity Survey.

### Baseline productivity within and outside the home

The burden of axSpA at study baseline was high, impacting workplace absenteeism and presenteeism as well as household productivity and participation in daily activities (Table 3).

**Table 3:** **Workplace and household productivity at baseline in axial spondyloarthritis**, **ankylosing spondylitis and nonradiographic axial spondyloarthritis populations (randomized sets, observed cases)** ^**a**^

	axSpA (***n*** = 324)			AS (***n*** = 178)			nr-axSpA (***n*** = 146)		
WPS questions ^b^	***n***	Mean (SD)	Median	***n***	Mean (SD)	Median	***n***	Mean (SD)	Median
Q2. Number of days of work missed (absenteeism)^c^	223	2.0 (4.8)	0.0	119	1.6 (3.5)	0.0	104	2.5 (6.0)	0.0
Q3. Number of days with productivity ≤50% at work (presenteeism)^c,d^	222	5.2 (7.6)	0.0	119	4.7 (7.1)	0.0	103	5.8 (8.0)	2.0
Q4. Rate of arthritis interference with work productivity^c,e^	222	4.6 (2.6)	5.0	119	4.5 (2.4)	5.0	103	4.8 (2.9)	5.0
Q5. Number of days of household work missed	324	5.8 (8.3)	2.0	178	5.2 (7.7)	1.0	146	6.5 (8.8)	3.0
Q6. Number of days with productivity ≤50% in household work^d^	324	7.5 (8.9)	5.0	178	7.0 (8.6)	4.0	146	8.1 (9.2)	5.0
Q7. Number of days of family, social or leisure activities missed	324	4.4 (6.9)	1.0	178	3.6 (6.1)	0.5	146	5.4 (7.8)	2.0
Q8. Number of days with outside help	324	2.1 (5.8)	0.0	178	1.6 (4.8)	0.0	146	2.7 (6.8)	0.0
Q9. Rate of arthritis interference with household work productivity^e^	324	4.9 (2.9)	5.0	178	4.9 (2.7)	5.0	146	4.8 (3.2)	5.0

^a^AS, Ankylosing spondylitis; axSpA, Axial spondyloarthritis, nr-axSpA, Nonradiographic axial spondyloarthritis. ^b^Recall period for Work Productivity Survey is 1 month. ^c^Assessed in employed patients only. ^d^Days counted exclude those counted in previous question (full days missed). ^e^Scored from 0 to 10, where 0 = no interference and 10 = complete interference.

Patients in jobs with some manual component had a higher number of workplace days missed per month than those in exclusively nonmanual jobs (mean 2.5 versus 1.4 days). Additionally, these patients reported more days per month with patient workplace productivity reduced by at least half compared to those with exclusively nonmanual jobs (mean 6.1 versus 4.3 days, respectively). In terms of household work, employed patients reported a high impact of axSpA symptoms, but the impact was lower compared to nonemployed patients and to patients unable to work due to arthritis (mean household work days missed per month: 4.6 versus 8.4 versus 14.6; mean days per month with household productivity reduced by ≥50%: 6.6 versus 9.4 versus 11.2, respectively).

### Completion rates of Work Productivity Survey at baseline

At baseline, all patients in the axSpA RS population answered at least one of the WPS questions. The completion rates of each of the WPS questions at baseline in the RS population were very high, indicating that the instrument was clear, acceptable and representative of the study population, and therefore that the results can be generalized to a larger axSpA population. There was only one (0.3%) missing answer to WPS Q5 to Q9 at baseline. Among all employed axSpA RS patients who were required to answer WPS Q2 through Q4, the completion rates at baseline were also high, with only two (0.9%) missing answers for Q2 and three (1.3%) missing answers for Q3 and Q4.

Similarly high completion rates of WPS questions were noted in the nr-axSpA and AS subpopulations. At baseline, there was no presence of a ceiling effect, as shown by the very small number of patients with a maximal answer. In the overall axSpA population, two (0.9%) to four (1.8%) of the RS employed patients had an answer ≥30 days to WPS Q2 and Q3, respectively, and ten (4.5%) had a maximum answer of 10 to Q4. Out of all RS patients, 5 (1.5%) to 21 (6.5%) had an answer ≥30 days to WPS Q5 to Q8 or a maximal score of 10 to Q9. Similarly, no ceiling effects were noted in the nr-axSpA and AS subpopulations.

As expected, in terms of floor effect, the percentage of patients with a minimal response varied between the WPS questions, with a higher number of patients answering 0 to Q2 (work days missed in the past month, 69.5% of the employed RS population in the overall axSpA population) and to Q8 (days with outside help hired, 78.1% of the entire RS population), and ranging from 11.3% to 50.5% for the other questions. Similar results were observed in the nr-axSpA and AS subpopulations.

### Discriminant validity

**Figure 1:**
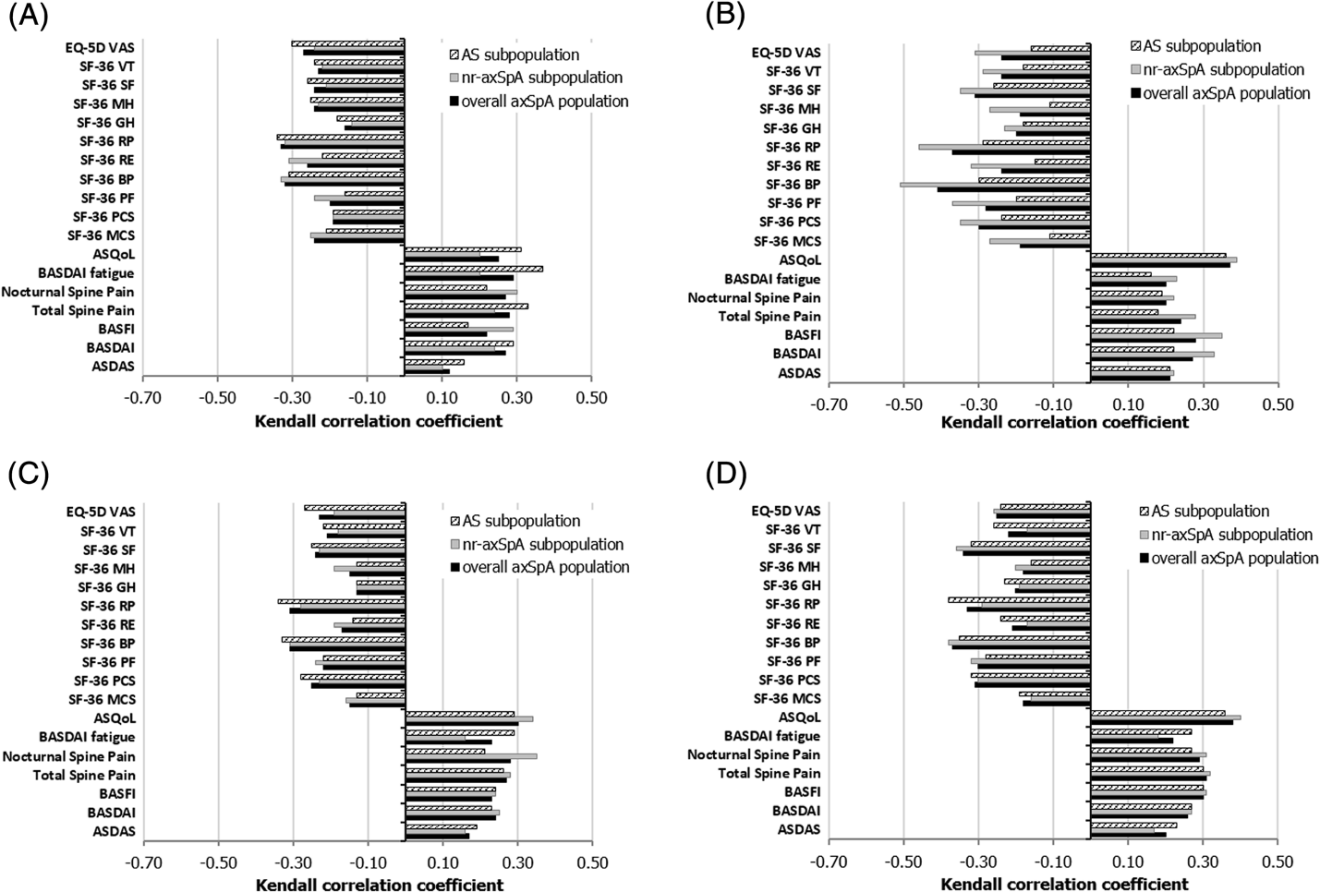
**Kendall association coefficients between Work Productivity Survey and clinical and health-related quality of life assessments at baseline (randomized set, observed cases).** Selective results of Kendall association coefficients are presented. **(A)** Work Productivity Survey (WPS) Question (Q)2 (number of work days missed because of arthritis). **(B)** WPS Q4 (rate of arthritis interference with work productivity. **(C)** WPS Q5 (number of days with no household work because of arthritis). **(D)** WPS Q9 (rate of arthritis interference with household work productivity). WPS Q2 and Q4 were assessed in employed patients only, whereas Q5 and Q9 were assessed in all patients. Q4 and Q9 are scored on a scale of 0 to 10 points (0 = no interference and 10 = complete interference). Correlation level (absolute value): low correlation (<0.3); moderate correlation (≥0.3 to <0.5). Ankylosing Spondylitis Disease Activity Score (ASDAS), Bath Ankylosing Spondylitis Functional Index (BASFI), Bath Ankylosing Spondylitis Disease Activity Index (BASDAI), Total/Nocturnal Spine Pain, Ankylosing Spondylitis Quality of Life Scale (ASQoL): lower score = better. Short Form 36 items (SF-36), EuroQoL 5 dimensions Visual Analogue Scale (EQ-5D VAS): higher score = better. WP: Work productivity.

The association coefficients between all WPS questions and different continuous measures assessing the disease activity, physical functioning and HRQoL, were low (<0.3) to moderate (≥0.3 to 0.5), as expected, indicating divergent validity between the individual WPS questions and the other measures considered (Figure 1).

The level and the sign (positive or negative) of the Kendall association coefficients indicated that better productivity at work and within the home (as assessed by lower scores to WPS Q2 to Q9) was associated with better HRQoL, less fatigue, better physical functioning or less pain (Figure 1). The range of the association coefficients between the individual WPS questions and the clinical and HRQoL assessments was similar in the overall axSpA, nr-axSpA and AS populations. Nevertheless, higher correlation coefficients were observed in nr-axSpA compared to AS between WPS Q4 (arthritis interference with work productivity (outside home)) and all clinical and HRQoL measures, as well as between Q8 (days with outside help hired) and certain HRQoL measures (SF-36 and ASQoL) (Figure 1).

The known-groups validity analysis indicated that there was a higher burden of arthritis on productivity at both paid work and within the home in patients with a worse health state compared to patients with a better health state (Tables 4 and 5). Among employed patients in the overall axSpA population, patients with a worse health state had higher workplace productivity losses, with significantly more work days lost and more work days with productivity reduced by half, and a statistically higher interference of arthritis on work productivity compared to patients with a better health state (Table 4). In the overall axSpA population, compared with patients with a better health state, patients with a worse health state had larger productivity losses within the household, with, on average, significantly more days of household work lost; more days with household productivity reduced by at least half; more days missed of family, social or leisure activities; more days with outside help hired; and a significantly higher interference of arthritis (all per month) (Table 5).

**Table 4:** **Work Productivity Survey baseline scores by defined known-groups: workplace productivity (randomized set, observed cases)** ^**a**^

Instrument ^b^	Number of days of work missed over the previous month, mean		Number of days with productivity ≤50% at work over the previous month, mean		Rate of arthritis interference with work productivity over previous month, mean ^c^	
	Worse	Better	Worse	Better	Worse	Better
BASDAI						
axSpA (cutoff 5.20 and 7.50)	3.27^d^	0.70	7.95^d^	2.86	5.44^d^	3.45
	*n* = 59	*n* = 57	*n* = 59	*n* = 57	*n* = 59	*n* = 57
AS (cutoff 5.20 and 7.30)	3.12^d^	0.56	8.12^d^	2.79	5.32^e^	3.71
	*n* = 34	*n* = 34	*n* = 34	*n* = 34	*n* = 34	*n* = 34
nr-axSpA (cutoff 5.40 and 7.60)	3.19^f^	0.79	9.63^f^	3.85	5.74^d^	3.26
	*n* = 27	*n* = 28	*n* = 27	*n* = 28	*n* = 27	*n* = 28
BASFI						
axSpA (cutoff 3.80 and 6.55)	4.02^d^	0.68	7.34^f^	4.22	5.79^d^	3.55
	*n* = 56	*n* = 56	*n* = 56	*n* = 56	*n* = 56	*n* = 56
AS (cutoff 4.30 and 6.80)	3.09^e^	0.87	5.47	2.73	5.31^d^	3.40
	*n* = 32	*n* = 30	*n* = 32	*n* = 30	*n* = 32	*n* = 30
nr-axSpA (cutoff 3.20 and 6.40)	6.04^d^	0.44	8.81	5.25	6.52^#^	3.63
	*n* = 27	*n* = 25	*n* = 27	*n* = 25	*n* = 27	*n* = 25
Total Spine Pain						
axSpA (cutoff 6 and 8)	2.97^e^	1.10	7.01^d^	2.99	5.57^d^	3.75
	*n* = 88	*n* = 82	*n* = 88	*n* = 82	*n* = 88	*n* = 82
AS (cutoff 6 and 8)	2.87^d^	0.52	6.40^e^	2.50	5.17^e^	3.90
	*n* = 47	*n* = 42	*n* = 47	*n* = 42	*n* = 47	*n* = 42
nr-axSpA (cutoff 6 and 8)	3.07	1.70	7.71^e^	3.51	6.02^d^	3.59
	*n* = 41	*n* = 40	*n* = 41	*n* = 40	*n* = 41	*n* = 40
SF-36 PCS						
axSpA (cutoff 37.54 and 27.49)	2.85^e^	0.76	6.13^f^*	2.98	5.85^d^	3.28
	*n* = 54	*n* = 55	*n* = 54	*n* = 55	*n* = 54	*n* = 55
AS (cutoff 37.44 and 27.93)	2.72^e^	0.62	6.48	2.86	5.62^d^	3.41
	*n* = 29	*n* = 29	*n* = 29	*n* = 29	*n* = 29	*n* = 29
nr-axSpA (cutoff 37.76 and 26.58)	3.84^f^	0.92	6.52	3.12	6.64^d^	3.12
	*n* = 25	*n* = 26	*n* = 25	*n* = 26	*n* = 25	*n* = 26
SF-36 MCS						
axSpA (cutoff 49.84 and 32.24)	4.78^d^	0.81	8.18^e^	3.54	5.58^e^	3.93
	*n* = 55	*n* = 80	*n* = 55	*n* = 80	*n* = 55	*n* = 80
AS (cutoff 52.73 and 32.87)	3.86^e^	0.89	7.00^f^	3.00	5.07	4.18
	*n* = 29	*n* = 28	*n* = 29	*n* = 28	*n* = 29	*n* = 28
nr-axSpA (cutoff 46.98 and 30.79)	5.65^d^	0.69	8.58^e^	3.23	6.08^e^	3.50
	*n* = 26	*n* = 26	*n* = 26	*n* = 26	*n* = 26	*n* = 26
ASQoL						
axSpA (cutoff 8.50 and 15.00)	4.40^d^	0.62	8.00^d^	3.35	6.26^d^	2.98
	*n* = 58	*n* = 90	*n* = 58	*n* = 90	*n* = 58	*n* = 90
AS (cutoff 8.00 and 14.91)	3.77^d^	0.42	7.53	3.61	5.73^d^	3.03
	*n* = 30	*n* = 31	*n* = 30	*n* = 31	*n* = 30	*n* = 31
nr-axSpA (cutoff 9.00 and 15.00)	5.07^d^	0.77	8.50^e^	3.33	6.82^d^	3.00
	*n* = 28	*n* = 30	*n* = 28	*n* = 30	*n* = 28	*n* = 30

^a^ASQoL: Ankylosing spondylitis quality of life; BASDAI: Bath Ankylosing Spondylitis Disease Activity Index; BASFI: Bath Ankylosing Spondylitis Functional Index; MCS: Mental Components Summary; PCS: Physical Components Summary; SF-36: Short form 36 items. ^b^Cutoff points represent the first and third quartiles of baseline scores: “Worse” state is defined for each individual measure as BASDAI score at or above the third quartile; BASFI score at or above the third quartile; Total Spine Pain score at or above the third quartile; ASQoL at or above the third quartile; SF-36 MCS at or below the first quartile; SF-36 PCS at or below the first quartile; “Better” state defined for each individual measure as BASDAI score at or below the first quartile; BASFI score at or below the first quartile; Total Spine Pain score at or below the first quartile; ASQoL score at or below the first quartile; SF-36 MCS, PCS at or above the third quartile; ^c^WPS Q4 is scored on a 0 to 10 scale, where 0 = no interference and 10 = complete interference. ^d^ *P* ≤ 0.001; ^e^ *P* ≤ 0.01; ^f^ *P* ≤ 0.05; nonparametric bootstrap-t method with a variance stabilizing transformation, 10,000 replications.

**Table 5:** **Work Productivity Survey baseline scores by defined known groups: household productivity and daily activities (randomized set, observed cases)** ^**a**^

Instrument ^b^	Number of days of household work missed over the previous month, mean		Number of days with household productivity ≤50% at work over the previous month, mean		Number of days of missed family, social, or leisure activities over the previous month, mean		Number of days with outside help over the previous month, mean		Rate of arthritis interference with household work productivity over previous month, mean ^c^	
	Worse	Better	Worse	Better	Worse	Better	Worse	Better	Worse	Better
BASDAI										
axSpA (cutoff 5.30 and 7.60)	9.36^d^	3.28	9.38^e^	5.44	6.05^d^	2.47	2.21^f^	0.78	6.00^d^	3.83
	*n* = 84	*n* = 86	*n* = 84	*n* = 86	*n* = 84	*n* = 86	*n* = 84	*n* = 86	*n* = 84	*n* = 86
AS (cutoff 5.20 and 7.60)	8.96^d^	3.04	8.39^f^	5.28	6.07^d^	2.30	1.72^f^	0.53	5.96^d^	3.77
	*n* = 46	*n* = 47	*n* = 46	*n* = 47	*n* = 46	*n* = 47	*n* = 46	*n* = 47	*n* = 46	*n* = 47
nr-axSpA (cutoff 5.40 and 7.60)	9.84^d^	3.38	10.58^f^	5.82	6.03^e^	2.51	2.82	1.08	6.05^d^	3.62
	*n* = 38	*n* = 39	*n* = 38	*n* = 39	*n* = 38	*n* = 39	*n* = 38	*n* = 39	*n* = 38	*n* = 39
BASFI										
axSpA (cutoff 3.80 and 7.00)	9.90^d^	2.95	9.24^f^	6.38	6.91^d^	2.71	3.01^e^	0.90	6.17^d^	3.56
	*n* = 87	*n* = 84	*n* = 87	*n* = 84	*n* = 87	*n* = 84	*n* = 87	*n* = 84	*n* = 87	*n* = 84
AS (cutoff 4.40 and 7.10)	10.66^d^	1.93	9.98^d^	4.56	6.68^d^	2.07	2.49^d^	0.38	6.45^d^	3.56
	*n* = 47	*n* = 45	*n* = 47	*n* = 45	*n* = 47	*n* = 45	*n* = 47	*n* = 45	*n* = 47	*n* = 45
nr-axSpA (cutoff 3.00 and 6.80)	9.13^f^	4.03	9.34	7.51	7.66^e^	2.78	3.53	1.70	6.16^d^	3.54
	*n* = 38	*n* = 37	*n* = 38	*n* = 37	*n* = 38	*n* = 37	*n* = 38	*n* = 37	*n* = 38	*n* = 37
Total Spine Pain										
axSpA (cutoff 6 and 8)	8.35^d^	2.54	8.51^d^	4.83	5.89^d^	3.03	2.06	1.78	6.09^d^	3.61
	*n* = 136	*n* = 109	*n* = 136	*n* = 109	*n* = 136	*n* = 109	*n* = 136	*n* = 109	*n* = 136	*n* = 109
AS (cutoff 6 and 8)	7.68^d^	2.36	8.03^e^	3.91	5.14^d^	1.54	1.42	1.45	6.00^d^	3.71
	*n* = 76	*n* = 56	*n* = 76	*n* = 56	*n* = 76	*n* = 56	*n* = 76	*n* = 56	*n* = 76	*n* = 56
nr-axSpA (cutoff 6 and 8)	9.20^d^	2.74	9.13^f^	5.81	6.83	4.60	2.87	2.13	6.20^d^	3.51
	*n* = 60	*n* = 53	*n* = 60	*n* = 53	*n* = 60	*n* = 53	*n* = 60	*n* = 53	*n* = 60	*n* = 53
SF-36 PCS										
axSpA (cutoff 37.35 and 27.30)	9.73^d^	2.23	10.85^d^	3.81	6.45^d^	2.13	3.23^d^	0.38	6.25^d^	3.28
	*n* = 80	*n* = 80	*n* = 80	*n* = 80	*n* = 80	*n* = 80	*n* = 80	*n* = 80	*n* = 80	*n* = 80
AS (cutoff 36.49 and 26.73)	9.55^d^	1.89	11.43^d^	4.30	5.61^e^	2.00	1.39	0.45	6.32^d^	3.43
	*n* = 44	*n* = 44	*n* = 44	*n* = 44	*n* = 44	*n* = 44	*n* = 44	*n* = 44	*n* = 44	*n* = 44
nr-axSpA (cutoff 38.25 and 27.40)	9.17^d^	2.61	9.72^d^	3.25	7.42^e^	2.28	5.11^d^	0.44	5.81^d^	3.00
	*n* = 36	*n* = 36	*n* = 36	*n* = 36	*n* = 36	*n* = 36	*n* = 36	*n* = 36	*n* = 36	*n* = 36
SF-36 MCS										
axSpA (cutoff 49.39 and 30.79)	8.88^d^	5.40	10.83^e^	6.69	8.63^d^	1.74	4.03^e^	1.28	5.94^d^	4.30
	*n* = 80	*n* = 80	*n* = 80	*n* = 80	*n* = 80	*n* = 80	*n* = 80	*n* = 80	*n* = 80	*n* = 80
AS (cutoff 49.93 and 31.38)	8.45^f^	4.61	10.25^f^	6.43	7.41^d^	1.30	2.52	1.00	6.14^e^	4.52
	*n* = 44	*n* = 44	*n* = 44	*n* = 44	*n* = 44	*n* = 44	*n* = 44	*n* = 44	*n* = 44	*n* = 44
nr-axSpA (cutoff 48.41 and 30.18)	10.36	6.36	11.64^f^	6.56	10.00^d^	2.28	5.39^f^	1.53	5.92^f^	4.19
	*n* = 36	*n* = 36	*n* = 36	*n* = 36	*n* = 36	*n* = 36	*n* = 36	*n* = 36	*n* = 36	*n* = 36
ASQoL										
axSpA (cutoff 9.00 and 15.00)	10.13^d^	2.66	10.41^d^	4.59	8.43^d^	1.29	4.21^d^	0.97	6.75^d^	3.32
	*n* = 97	*n* = 90	*n* = 97	*n* = 90	*n* = 97	*n* = 90	*n* = 97	*n* = 90	*n* = 97	*n* = 90
AS (cutoff 9.00 and 15.00)	9.00^d^	2.30	10.49^d^	4.30	6.96^d^	1.43	3.30^d^	0.55	6.42^d^	3.47
	*n* = 53	*n* = 47	*n* = 53	*n* = 47	*n* = 53	*n* = 47	*n* = 53	*n* = 47	*n* = 53	*n* = 47
nr-axSpA (cutoff 9.00 and 15.00)	11.50^d^	3.05	10.32^e^	4.91	10.20^d^	1.14	5.30^f^	1.42	7.16^d^	3.16
	*n* = 44	*n* = 43	*n* = 44	*n* = 43	*n* = 44	*n* = 43	*n* = 44	*n* = 43	*n* = 44	*n* = 43

^a^ASQoL: Ankylosing spondylitis quality of life; BASDAI: Bath Ankylosing Spondylitis Disease Activity Index; BASFI: Bath Ankylosing Spondylitis Functional Index; MCS: Mental Components Summary; PCS: Physical Components Summary; SF-36: Short form 36 items. ^b^Cutoff points represent the first and third quartiles of baseline scores: “Worse” state is defined for each individual measure as BASDAI score at or above the third quartile; BASFI score at or above the third quartile; Total Back Pain score at or above the third quartile; ASQoL at or above the third quartile; SF-36 MCS at or below the first quartile; SF-36 PCS at or below the first quartile; “better” state is defined for each individual measure as BASDAI score at or below the first quartile; BASFI score at or below the first quartile; Total Back Pain score at or below the first quartile; ASQoL score at or below the first quartile; SF-36 MCS, PCS at or above the third quartile. ^c^WPS Q4 is scored on a 0 to 10 scale, where 0 = no interference and 10 = complete interference. ^d^ *P* ≤ 0.001; ^e^ *P* ≤ 0.01; ^f^ *P* ≤ 0.05; nonparametric bootstrap-t method with a variance stabilizing transformation, 10,000 replications.

Similar findings were observed in the nr-axSpA and AS subpopulations, where patients with a worse health state had a higher burden of arthritis on productivity, at both paid work and within home, compared to patients with a better health state (Tables 4 and 5).

### Responsiveness and reliability

#### Work Productivity Survey changes from baseline by SpondyloArthritis International Society (ASAS) 20% improvement criteria response at week 12

**Figure 2:**
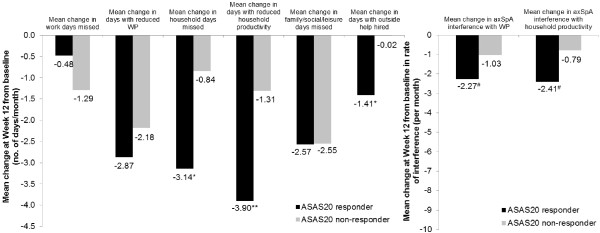
**Mean changes from baseline in Work Productivity Survey by SpondyloArthritis International Society 20% improvement criteria clinical response at week 12.** Change from baseline in Work Productivity Survey (WPS) by Assessment of Ankylosing Spondyloarthritis International Society 20% improvement criteria (ASAS20) clinical response at week 12 in overall axial spondyloarthritis (axSpA) population (randomized set, observed cases). *P ≤ 0.05, **P ≤ 0.01, ^#^ *P* ≤ 0.001; responders versus nonresponders. *P*-values were obtained using the nonparametric bootstrap-t method. Rate of interference is a score on a scale of 0 to 10 points (0 = no interference and 10 = complete interference). WP: Work productivity.

Significantly larger improvements in productivity within and outside the home (corresponding to higher negative mean changes in WPS responses) were reported by ASAS20 responders at week 12 compared to ASAS20 nonresponders in the overall axSpA population, except with regard to absenteeism, presenteeism and days missed of family, social or leisure activities, where only numerical differences were seen (Figure 2). Although differences in changes in absenteeism were noted between ASAS20 responders and nonresponders, the level of improvements in the nonresponders was numerically greater than the level of changes in responders, which might be explained by the difference in the baseline scores between the two groups (mean 3.1 days per month missed at baseline versus 1.4 days per month, respectively).

Similarly, in the AS and nr-axSpA subgroups, patients achieving an ASAS20 response at week 12 reported greater improvements in productivity, both within and outside the home, compared to baseline. As in the overall axSpA population, differences in absenteeism seemed to favor nonresponders over responders in the AS and nr-axSpA populations; however, this may be explained by the differences in baseline productivity loss.

**Figure 3:**
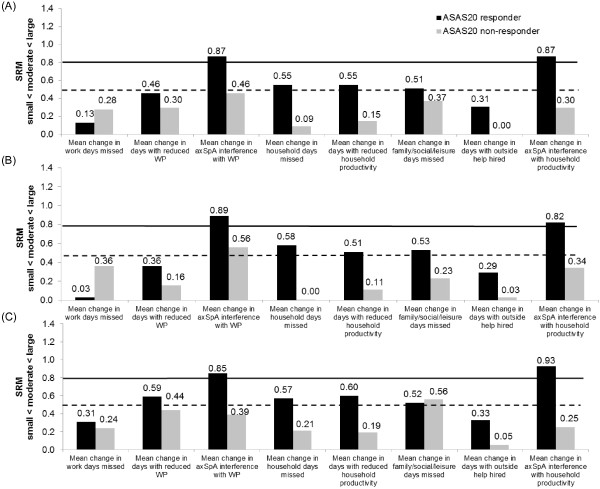
**Effect size of mean changes from baseline in Work Productivity Survey by SpondyloArthritis International Society 20% improvement criteria clinical response at week 12.** Effect size (standardized response mean, SRM) of mean changes from baseline in Work Productivity Survey (WPS) are presented by Assessment of Ankylosing Spondyloarthritis International Society 20% improvement criteria (ASAS20) clinical response at week 12 for overall axial spondyloarthritis (axSpA) population **(A)**, ankylosing spondyloarthritis (AS) subpopulation **(B)** and nonradiographic axial spondyloarthritis (nr-axSpA) subpopulation **(C)** (randomized set, observed cases). SRM (absolute values) thresholds: small (from 0.2 to 0.5; below the dashed line); moderate (from 0.5 to 0.8; between the two lines) and large (>0.8; above the solid line). WP: Work productivity.

For axSpA patients, the effect sizes of the changes in productivity, measured by the SRM, in ASAS20 responders were small (SRM < 0.5) for absenteeism, presenteeism and days with outside help, but moderate to large for the other WPS questions. In nonresponders, the magnitude of change was negligible (SRM < 0.1) or small (SRM < 0.5) (Figure 3). Similar findings were found in the nr-axSpA and AS subpopulations.

#### Work Productivity Survey changes from baseline by other response measures at week 12

The responsiveness of the WPS when using the BASDAI50 response criteria resulted in similar findings in the overall axSpA, nr-axSpA and AS populations. In all three populations, BASDAI50 responders at week 12 reported significantly or numerically greater improvements compared to nonresponders, except in absenteeism (in axSpA and nr-axSpA populations) and days missed of social activities (nr-axSpA subpopulation), where slightly higher improvements were noticed in nonresponders versus responders; however, this may be explained by the differences in baseline productivity losses (data not shown). The pattern of productivity change effect sizes observed in BASDAI50 responders and nonresponders were similar to those observed in ASAS20 responders and nonresponders.

With regard to the responsiveness of WPS to more stringent clinical responses, such as ASAS40 or ASAS50, the effect sizes in mean changes in productivity within and outside the home in the responder groups were moderate to large, except for two questions (absenteeism and days with outside help) for which the effect sizes were small (SRM < 0.5). Results were similar in the overall axSpA population, nr-axSpA and AS subpopulations, except for absenteeism and days missed of social activities, which indicated different behaviors in the nr-axSpA and AS subpopulations (data not shown).

The results based on the total and nocturnal back pain MCID response were inconclusive because of a large imbalance in the sample sizes of the two groups compared.

**Figure 4:**
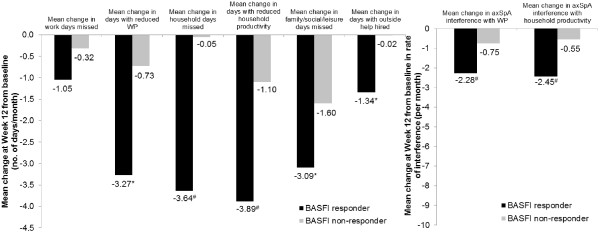
**Mean changes from baseline in Work Productivity Survey by Bath Ankylosing Spondylitis Functional Index response at week 12.** Change from baseline in Work Productivity Survey (WPS) by Bath Ankylosing Spondylitis Functional Index (BASFI) response at week 12 in overall axial spondyloarthritis (axSpA) population (randomized set, observed cases). *P ≤ 0.05, #*P* ≤ 0.001, responders versus nonresponders. *P*-values were obtained using the nonparametric bootstrap-t method. Rate of interference is a score on a scale of 0 to 10 points (0 = no interference and 10 = complete interference). WP: Work productivity.

**Figure 5:**
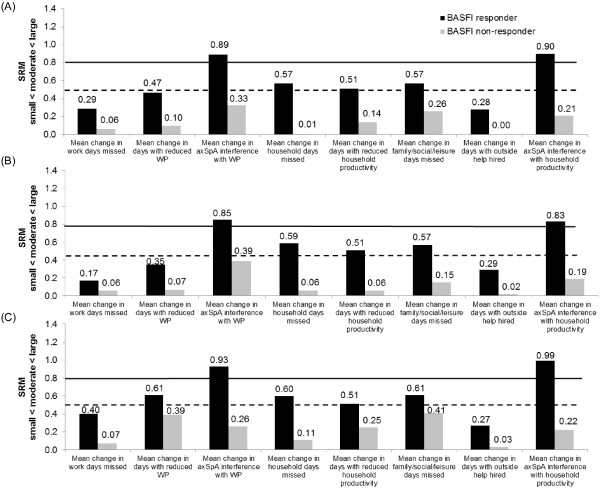
**Effect size of mean changes from baseline in Work Productivity Survey by Bath Ankylosing Spondylitis Functional Index response at week 12.** Effect size (standardized response mean, SRM) of mean changes from baseline in Work Productivity Survey (WPS) are presented by Bath Ankylosing Spondylitis Functional Index (BASFI) response at week 12 for overall axial spondyloarthritis (axSpA) population **(A)**, ankylosing spondyloarthritis (AS) subpopulation **(B)** and nonradiographic axial spondyloarthritis (nr-axSpA) subpopulation **(C)** (randomized set, observed cases). SRM (absolute values) thresholds: small (from 0.2 to 0.5; below the dashed line); moderate (from 0.5 to 0.8; between the two lines) and large (>0.8; above the solid line). WP: Work productivity.

With regard to productivity changes were compared between responders and nonresponders at week 12 defined using other clinical response criteria, such as the ASDAS CII, ASDAS MI or the BASFI MCID response (Figure 4), clinical responders reported significantly or numerically larger improvements in productivity within and outside the home across all WPS questions in all three populations. In the overall axSpA population, the effect sizes of the changes in productivity in ASDAS CII, ASDAS MI or BASFI responders (Figure 5) were small (SRM < 0.5) for absenteeism (WPS Q2) and days with outside help (WPS Q8), and generally moderate to large for the other WPS questions. In nonresponders, the magnitude of change was negligible (SRM < 0.1) or small (SRM < 0.5). Similar effect sizes were observed in the nr-axSpA and AS subpopulations (data not shown).

## Discussion

This work assessed the initial psychometric properties of the WPS, originally developed in a population of patients with RA [17], in patients with axSpA, nr-axSpA and AS. The disease specific WPS is a tool developed to estimate productivity limitations in the workplace and relating to household activities due to arthritis [17] and whose psychometric properties have already been demonstrated in patients with active RA [17]. Previous work demonstrated that the WPS could efficiently evaluate both the burden of the disease and clinical interventions on work outcomes in patients with RA [17, 32, 33].

The discriminant validity, responsiveness to clinical changes and reliability of the survey were evaluated in patients enrolled in a clinical trial for the treatment of active axSpA. The OMERACT meetings 6 and 7 [34, 35] highlighted the importance to patients of consideration of the impact of arthritic conditions on paid and unpaid work outcomes, as these factors represent an important component of the health and well-being of rheumatology patients. Similar thinking should apply to patients with axSpA. Patient-reported outcomes have long been included in rheumatology trials, as they capture the patient’s perspective of the disease process and the impact of treatments on the disease. Despite being of interest to patients and employers, the impact of axSpA or AS on work outcomes is not currently a core component of rheumatology clinical trials.

The spondyloarthritis treatment guidelines of the Canadian Rheumatology Association and the Spondyloarthritis Research Consortium of Canada indicate that disease monitoring should include assessments of function, disability and handicap and further noted that “[p]articipation in social, leisure, education, community and work activities must be an integral measure used to evaluate outcomes by health professionals, educators, policymakers and researchers” 2279 [36]. Furthermore, the ASAS has indicated the importance of worker productivity in its educational slides. OMERACT has reinforced the importance of work productivity as an outcome measure in rheumatology through the Worker Productivity Special Interest Group, which has reviewed specific productivity instruments and continues to evaluate concepts and methodological and interpretation issues in work productivity [37].

The present findings indicate that the WPS instrument was generally well understood by patients, as indicated by the high completion rates. As in rheumatoid arthritis, the WPS demonstrated good discriminant validity, in terms of both association coefficients and known-groups analyses, evaluated against a range of different continuous measures used to assess disease activity, physical functioning and HRQoL. The association coefficients indicate the divergent validity between the individual WPS questions and the other measures considered, which was further confirmed by the known-groups analyses. Findings in the nr-axSpA and AS subpopulations were similar to those in the overall axSpA population.

The known-groups analyses based on the first and third quartiles of the instrument scores at baseline were further confirmed using a median cutoff of the score. However, the responsiveness of the WPS was assessed using clinically recognized thresholds and supports the discriminant validity analysis.

The WPS was also responsive to clinical changes, as measured by the ASAS20 and BASDAI50 responses at week 12. Findings were also similar when using a range of different clinical response measures (ASAS40, ASAS50, ASDAS MI, ASDAS CII and BASFI MCID), supporting the responsiveness and reliability of the WPS. Similar results were reported in all three populations (axSpA, nr-axSpA and AS).

All WPS questions showed a certain level of responsiveness to clinical changes. However, the responsiveness of questions concerning the number of work days missed due to axSpA and the number of days with outside help hired were not as large or consistent as the other WPS questions. The number of respondents who actually reported full days of work missed, or days with outside help, was quite small relative to the entire study sample. The results for absenteeism might suggest that the impact of axSpA on productivity more likely manifests as daily interference with normal working practices, without resulting in full disability. However, it should also be noted that because 12.4% of the sample reported being unable to work due to arthritis at baseline, patients who might otherwise have reported missing a high number of days of work did not report their level of absenteeism. The low level of responsiveness for the question assessing days with hired outside help would appear to suggest that axSpA patients might not necessarily hire outside help, but the possibility that patients receive external help from relatives or friends cannot be excluded.

Given the intent of using the WPS across a variety of rheumatic conditions, including those where higher levels of disability might be anticipated, all questions of the WPS should remain to ensure an accurate assessment of the impact of arthritis on different aspects of work and household productivity.

The responsiveness and reliability of the WPS for use in axSpA has been confirmed not only by the differences in the mean changes in WPS scores but also by the effect sizes seen for responders and nonresponders and the similarities in responsiveness when using different response criteria. This was true for the overall axSpA population as well as the nr-axSpA and AS subpopulations.

Although there is no gold standard for assessing worker productivity in axSpA, these results are in line with previous work carried out for the Work Productivity and Activity Impairment (WPAI) questionnaire in the AS subpopulation. Similar to the current findings in the AS subpopulation, in the previous work carried out using the WPAI, patients with greater disease severity (assessed using BASDAI) experienced greater work impairment, presenteeism and impairment in daily activities; however, absenteeism was not significantly different between patients with worse health compared to those with better health [38].

The limitations of this study include the patient population, which was recruited for a clinical trial of active axSpA and therefore may not be completely representative of the wider axSpA population. However, as the WPS is currently developed as a tool for clinical trials, the patient population used should provide sufficient evidence to ensure the validity of this measure in its current role. The WPS is also affected by the normal limitations of self-reported questionnaires; however, previous reports have confirmed that this is still the best means of collecting such data [39].

## Conclusions

The WPS was found to be a valid instrument in axSpA, nr-axSpA and AS on the basis of its ability to discriminate between patients with different disease symptom severity and between patients who respond to recognized clinical changes. This survey can be used to capture the impact of active axSpA on productivity in the workplace and within the household, as well as participation in daily activities. It is also an informative instrument for use in clinical trials and in clinical practice, enabling assessment of the impact of treatment on axSpA-related workplace and household productivity losses.

## Electronic supplementary material

Additional file 1: **Ethical bodies.** List of ethical bodies approving RAPID-PsA study. (PDF 977 KB)

Additional file 2: **Questionnaires assessed.** Details of the questionnaires assessed in the article. (DOCX 29 KB)

## Abbreviations

AS: Ankylosing spondylitis
ASAS: Assessment of SpondyloArthritis International Society
ASAS 20: ASAS 20% improvement criteria
ASDAS: Ankylosing Spondylitis Disease Activity Score
ASQoL: Ankylosing Spondylitis Quality of Life Scale
axSpA: Axial spondyloarthritis
BASDAI: Bath Ankylosing Spondylitis Disease Activity Index
BASFI: Bath Ankylosing Spondylitis Functional Index
CII: Clinically important improvement
CRP: C-reactive protein
CZP: Certolizumab pegol
EQ-5D: EuroQoL 5-dimensions
HLA: Human leukocyte antigen
HRQoL: Health-related quality of life
MCID: Minimum clinically important difference
MCS: Mental Component Summary
MI: Major improvement
nr-axSpA: Nonradiographic axial spondyloarthritis
NRS: Numerical Rating Scale
PCS: Physical Component Summary
PtGADA: Patient’s Global Assessment of Disease Activity
Q: Question
Q2W: Every 2 weeks
Q4W: Every 4 weeks
RA: Rheumatoid arthritis
RS: Randomized set
SF-36: Short Form 36-items
SRM: Standardized response mean
VAS: Visual Analogue Scale
WPS: Work Productivity Survey.
