# Supplementary material for: Discriminant validity, responsiveness and reliability of the arthritis-specific Work Productivity Survey assessing workplace and household productivity within and outside the home in patients with axial spondyloarthritis, including nonradiographic axial spondyloarthritis and ankylosing spondylitis
Source: Arthritis Res Ther. 2014 Aug 6;16(4):R164. doi: 10.1186/ar4680 (PMC4448884; doi:10.1186/ar4680)
Supplement: Supplementary file 1 — Additional file 1: Ethical bodies. List of ethical bodies approving RAPID-PsA study. (PDF 977 KB) [file 13075_2013_4379_MOESM1_ESM.pdf]

LIST OF INSTITUTIONAL REVIEW BOARDS/INDEPENDENT ETHICS COMMITTEES

Site number	Principal investigator	IRB/IEC name, address, and committee chairperson
Central IEC<br>Belgium		Commissie Medische Ethiek<br>UZ Leuven campus Gasthuisberg<br>Herestraat 49<br>3000 Leuven<br>BELGIUM<br>Chairperson: Prof. W. Van den Bogaert, MD
150	de Vlam MD, Kurt<br>(did not enroll)	See above; Commissie Medische Ethiek UZ Leuven campus Gasthuisberg
151	Malaise MD, Michel	See above; Commissie Medische Ethiek UZ Leuven campus Gasthuisberg
152	van den Bosch MD, Filip	See above; Commissie Medische Ethiek UZ Leuven campus Gasthuisberg
153	Margaux MD, Joëlle	See above; Commissie Medische Ethiek UZ Leuven campus Gasthuisberg
Central IEC<br>France	--	Comité de Protection des Personnes (CPP)<br>Ile de France VIII<br>Hôspital Ambrose Paré<br>Laboratoire d'Anatomopathologie<br>9, avenue Charles-de-Gaulle<br>92100 Boulogne-Billancourt<br>FRANCE<br>Chairperson: Frédérique Barthod, MD
200	Breban MD, Maxime	See above; Comité de Protection des Personnes (CPP) Ile de France VIII

Site number	Principal investigator	IRB/IEC name, address, and committee chairperson
201	Flipo MD, René-Marc<br>(did not enroll)	See above; Comité de Protection des Personnes (CPP) Ile de France VIII
202	Goupille MD, Philippe	See above; Comité de Protection des Personnes (CPP) Ile de France VIII
204	Fautrel MD, Bruno	See above; Comité de Protection des Personnes (CPP) Ile de France VIII
205	Bertin MD, Philippe<br>(did not enroll)	See above; Comité de Protection des Personnes (CPP) Ile de France VIII
206	Combe MD, Bernard<br>(did not enroll)	See above; Comité de Protection des Personnes (CPP) Ile de France VIII
Central IEC<br>Germany	--	Ethik-Kommission der Ärztekammer Westfalen-Lippe und der Medizinischen Fakultät der Westfälischen Wilhelms-Universität Münster<br>Von-Esmarch-Str. 62<br>48149 Münster<br>GERMANY<br>Chairperson: Prof. Dr. Hans-Werner Bothe, MA
Central IEC<br>Germany	--	Ethik-Kommission des Fachbereichs Medizin der Johann Wolfgang Goethe-Universität Frankfurt<br>Theodor-Stern-Kai 7<br>60590 Frankfurt am Main<br>GERMANY<br>Chairperson: Prof. Dr. med. Sebastian Harder

Site number	Principal investigator	IRB/IEC name, address, and committee chairperson
Central IEC<br>Germany	--	Ethik-Kommission des Landes Berlin<br>Fehrbelliner Platz 1<br>10707 Berlin<br>GERMANY<br>Chairperson: PD Dr. med. K. Thomas Moesta
Central IEC<br>Germany	--	Ethik-Kommission der Medizinischen Fakultät der<br>Ludwig-Maximilians Universität, München<br>Pettenkoferstr. 8a<br>80336 München<br>GERMANY<br>Chairperson: Prof. Dr. Wolfgang Eisenmenger
250	Braun MD, Jürgen	See above; Ethik-Kommission der Ärztekammer Westfalen-Lippe und der Medizinischen Fakultät der Westfälischen Wilhelms-Universität Münster
252	Müller-Ladner MD, Ulf<br>(did not enroll)	See above; Ethik-Kommission der Ärztekammer Westfalen-Lippe und der Medizinischen Fakultät der Westfälischen Wilhelms-Universität Münster;<br><br>See above; Ethik-Kommission des Fachbereichs Medizin der Johann Wolfgang Goethe-Universität Frankfurt

Site number	Principal investigator	IRB/IEC name, address, and committee chairperson
253	Stahl MD, Hans-Detlev<br>(replaced by Elizaveta Degtyareva, MD)<br>Degtyareva MD, Elizaveta<br>(replaced Hans-Detlev Stahl, MD)	See above; Ethik-Kommission der Ärztekammer Westfalen-Lippe und der Medizinischen Fakultät der Westfälischen Wilhelms-Universität Münster;<br><br>Ethikkommission bei der Sächsischen Landesärztekammer<br>Schützenhöhe 16<br>01099 Dresden<br>GERMANY<br>Chairperson: Prof. Dr. med. Rolf Haupt
254	Wollenhaupt MD, Jürgen	See above; Ethik-Kommission der Ärztekammer Westfalen-Lippe und der Medizinischen Fakultät der Westfälischen Wilhelms-Universität Münster;<br><br>Ethik-Kommission der Ärztekammer Hamburg<br>Humboldtstr. 67 a<br>22083 Hamburg<br>GERMANY<br>Chairperson: Prof. Dr. med. Thomas Weber
255	Bannert MD, Bettina<br>(did not enroll)	See above; Ethik-Kommission der Ärztekammer Westfalen-Lippe und der Medizinischen Fakultät der Westfälischen Wilhelms-Universität Münster;<br><br>Ethik-Kommission der Albert-Ludwigs-Universität Freiburg<br>Engelberger Str. 1<br>79106 Freiburg<br>GERMANY<br>Chairperson: Prof. Dr. Drs. Hc Stefan Pollak

Site number	Principal investigator	IRB/IEC name, address, and committee chairperson
256	Wassenberg MD, Siegfried	See above; Ethik-Kommission der Ärztekammer Westfalen-Lippe und der Medizinischen Fakultät der Westfälischen Wilhelms-Universität Münster;<br><br>Ethik-Kommission der Ärztekammer Nordrhein<br>Tersteegenstr. 31<br>40474 Düsseldorf<br>GERMANY<br>Chairperson: Prof. Dr. Ursula Sehrt-Ricken (Essen)
257	Sieper MD, Joachim	See above; Ethik-Kommission der Ärztekammer Westfalen-Lippe und der Medizinischen Fakultät der Westfälischen Wilhelms-Universität Münster;<br><br>See above; Ethik-Kommission des Landes Berlin
258	Brandt-Jürgens MD, Jan	See above; Ethik-Kommission der Ärztekammer Westfalen-Lippe und der Medizinischen Fakultät der Westfälischen Wilhelms-Universität Münster;<br><br>See above; Ethik-Kommission des Landes Berlin

Site number	Principal investigator	IRB/IEC name, address, and committee chairperson
259	Gauler MD, Georg<br>(did not enroll)	See above; Ethik-Kommission der Ärztekammer Westfalen-Lippe und der Medizinischen Fakultät der Westfälischen Wilhelms-Universität Münster;<br><br>Ethik-Kommission der Ärztekammer Niedersachsen<br>Ethik-Kommission zur Beurteilung medizinischer Forschung am Menschen<br>Berliner Allee 20<br>30175 Hannover<br>GERMANY<br>Chairperson: Prof. Dr. Peter Kleine, Dr. jur.
260	Witt MD, Matthias<br>(did not enroll)	See above; Ethik-Kommission der Ärztekammer Westfalen-Lippe und der Medizinischen Fakultät der Westfälischen Wilhelms-Universität Münster;<br><br>See above; Ethik-Kommission der Medizinischen Fakultät der Ludwig-Maximilians Universität, München
262	Behrens MD, Frank<br>(did not enroll)	See above; Ethik-Kommission der Ärztekammer Westfalen-Lippe und der Medizinischen Fakultät der Westfälischen Wilhelms-Universität Münster;<br><br>See above; Ethik-Kommission des Fachbereichs Medizin der Johann Wolfgang Goethe-Universität Frankfurt
263	Kellner MD, Herbert	See above; Ethik-Kommission der Ärztekammer Westfalen-Lippe und der Medizinischen Fakultät der Westfälischen Wilhelms-Universität Münster;<br><br>See above; Ethik-Kommission der Medizinischen Fakultät der Ludwig-Maximilians Universität, München

Site number	Principal investigator	IRB/IEC name, address, and committee chairperson
Central IEC<br>Hungary	--	Egészségügyi Tudományos Tanács Klinikai Farmakológiai Etikai Bizottsága<br>Arany János. u. 6-8<br>1051 Budapest<br>HUNGARY<br>Chairperson: Dr. Susanna Fürst
300	Drescher MD, Edit	See above; Egészségügyi Tudományos Tanács Klinikai Farmakológiai Etikai Bizottsága
301	Keszthelyi MD, Péter<br>(did not enroll)	See above; Egészségügyi Tudományos Tanács Klinikai Farmakológiai Etikai Bizottsága
302	Szántó MD, Sándor	See above; Egészségügyi Tudományos Tanács Klinikai Farmakológiai Etikai Bizottsága
303	Újfalussy MD, Ilona	See above; Egészségügyi Tudományos Tanács Klinikai Farmakológiai Etikai Bizottsága
305	Géher MD, Pál	See above; Egészségügyi Tudományos Tanács Klinikai Farmakológiai Etikai Bizottsága
306	Tamási MD, László	See above; Egészségügyi Tudományos Tanács Klinikai Farmakológiai Etikai Bizottsága
350	Bombardieri MD, Stefano	Comitato Etico per la Sperimentazione Clinica dei Medicinali dell'Azienda Ospedaliera Universitaria Pisani di Pisa<br>Via Roma, 67<br>56126 Pisa<br>ITALY<br>Chairperson: Prof. Romano Danesi

Site number	Principal investigator	IRB/IEC name, address, and committee chairperson
351	Matucci Cerinic MD, Marco	Comitato Etico per la Sperimentazione Clinica dei Medicinali dell'Azienda Ospedaliera-Universitaria Careggi<br>Largo Brambilla, 3<br>50134 Firenze<br>ITALY<br>Chairperson: Dr. ssa Raffaella Giardiello
352	Grassi MD, Walter<br>(did not enroll)	Comitato Etico dell'Azienda Sanitaria Unica Regionale Delle Marche Di Ancona<br>Via Caduti del Lavoro, 40<br>60131 Ancona<br>ITALY<br>Chairperson: Dott. Gilberto Gentili
Central IEC<br>The Netherlands	--	Medische-Ethische Commissie academisch ziekenhuis Maastricht en Maastricht University (METC azM/UM)<br>P. Debyelaan 25<br>Postbus 5800<br>6202 AZ Maastricht<br>THE NETHERLANDS<br>Chairperson: Mw. Dr. CED de Die-Smulders
400	Han MD, K. H.<br>(did not enroll)	See above; Medische-Ethische Commissie academisch ziekenhuis Maastricht en Maastricht University (METC azM/UM)
401	Landewé MD, Robert<br>(replaced by Astrid van Tubergen, MD)<br>van Tubergen MD, Astrid<br>(replaced Robert Landewé, MD)	See above; Medische-Ethische Commissie academisch ziekenhuis Maastricht en Maastricht University (METC azM/UM)

Site number	Principal investigator	IRB/IEC name, address, and committee chairperson
Central IEC<br>Poland	--	Komisja Bioetyczna przy Okregowej Izbie Lekarskiej w Krakowie<br>Krupnicza 11a<br>31-123 Krakow<br>POLAND<br>Chairperson: kol. Stefan Bednarz
450	Chudzik MD, Dariusz	See above; Komisja Bioetyczna przy Okregowej Izbie Lekarskiej w Krakowie
452	Hrycaj MD, Pawel	See above; Komisja Bioetyczna przy Okregowej Izbie Lekarskiej w Krakowie
453	Jeka MD, Slawomir	See above; Komisja Bioetyczna przy Okregowej Izbie Lekarskiej w Krakowie
454	Leszczynski MD, Piotr	See above; Komisja Bioetyczna przy Okregowej Izbie Lekarskiej w Krakowie
455	Brzezicki MD, Jan	See above; Komisja Bioetyczna przy Okregowej Izbie Lekarskiej w Krakowie
456	Dudek MD, Anna	See above; Komisja Bioetyczna przy Okregowej Izbie Lekarskiej w Krakowie
457	Korkosz MD, Mariusz	See above; Komisja Bioetyczna przy Okregowej Izbie Lekarskiej w Krakowie
458	Racewicz MD, Artur	See above; Komisja Bioetyczna przy Okregowej Izbie Lekarskiej w Krakowie
459	Smolenska MD, Zaneta	See above; Komisja Bioetyczna przy Okregowej Izbie Lekarskiej w Krakowie
461	Brzosko MD, Marek<br>(did not enroll)	See above; Komisja Bioetyczna przy Okregowej Izbie Lekarskiej w Krakowie
462	Rell-Bakalarska MD, Maria	See above; Komisja Bioetyczna przy Okregowej Izbie Lekarskiej w Krakowie

Site number	Principal investigator	IRB/IEC name, address, and committee chairperson
463	Zajdel MD, Jerzy<br>(did not enroll)	See above; Komisja Bioetyczna przy Okregowej Izbie Lekarskiej w Krakowie
Central IEC<br>Czech Republic	--	Etická komise IKEM a FTNsP<br>Fakultní Thomayerova nemocnice s polikfinikou<br>Videnská 800<br>140 59 Praha 4 – Krc<br>CZECH REPUBLIC<br>Chairperson: Prof. MUDr. Vladimír Stanék, CSc.
Central IRC<br>Czech Republic	--	Etická komise<br>Revmatologický ústav<br>Na Slupi 4<br>128 50 Praha 2<br>CZECH REPUBLIC<br>Chairperson: RNDR. Ivana Pútová
500	Dvorák MD, Zdenek	See above; Etická komise IKEM a FTNsP, Fakultní Thomayerova nemocnice s polikfinikou;<br><br>See above; Etická komise Revmatologický ústav
501	Novosad MD, Libor	See above; Etická komise IKEM a FTNsP, Fakultní Thomayerova nemocnice s polikfinikou;<br><br>See above; Etická komise Revmatologický ústav
502	Vencovský MD, Jirí	See above; Etická komise IKEM a FTNsP, Fakultní Thomayerova nemocnice s polikfinikou;<br><br>See above; Etická komise Revmatologický ústav

Site number	Principal investigator	IRB/IEC name, address, and committee chairperson
503	Vítek MD, Petr	See above; Etická komise IKEM a FTNsP, Fakultní Thomayerova nemocnice s polikfinikou;<br><br>See above; Etická komise Revmatologický ústav
504	Némec MD, Petr	See above; Etická komise IKEM a FTNsP, Fakultní Thomayerova nemocnice s polikfinikou;<br><br>See above; Etická komise Revmatologický ústav
505	Záhora MD, Roman	See above; Etická komise IKEM a FTNsP, Fakultní Thomayerova nemocnice s polikfinikou;<br><br>See above; Etická komise Revmatologický ústav
Central IEC<br>Spain	--	Comité Ético de Investigación Clinica de Galicia<br>Unidad: Subdirección Xeral de Farmacia e Produtos Sanitarios. Conselleria de Sanidade<br>San Lázaro, s/n<br>15703 Santiago Compostela (La Coruña)<br>SPAIN<br>Chairperson: Dr. Rosendo Bugarín González
Central IEC<br>Spain	--	CEIC Autonómico de Ensayos<br>Clinicos de Andalucía<br>Conserjería de Salud<br>Avenida de la Innovación, s/n<br>Edificio Arena 1<br>41020 Sevilla<br>SPAIN<br>Chairperson: Dr. Demetrio Mariano Aguayo Canela

Site number	Principal investigator	IRB/IEC name, address, and committee chairperson
550	Chamizo Carmona MD, Eugenio<br>(did not enroll)	See above; Comité Ético de Investigación Clinica de Galicia<br>Unidad: Subdirección Xeral de Farmacia e Produtos<br>Sanitarios. Conselleria de Sanidade;<br><br>CEIC Hospital Universitario Infanta Cristina<br>Departamento de Farmacología de la Facultad de Medicina<br>Avenida de Elvas, s/n<br>06008 Badajoz<br>SPAIN<br>Chairperson: Dra. Francisca Lourdes Márquez Pérez
551	Collantes-Estévez MD, Eduardo<br>(did not enroll)	See above; Comité Ético de Investigación Clinica de Galicia<br>Unidad: Subdirección Xeral de Farmacia e Produtos<br>Sanitarios. Conselleria de Sanidade;<br><br>See above; CEIC Autonómico de Ensayos, Clinicos de Andalucía, Conserjería de Salud;<br><br>Secretaría Técnica del CEIC<br>Hospital Universitario Reina Sofia<br>Edificio Consultas Externas pl-1<br>Avenida Menéndez Pidal, s/n<br>14004 Córdoba<br>SPAIN<br>Chairperson: Dr. Jose Luis Barranco Quintana
552	Gómez-Reino MD, Juan J.<br>(did not enroll)	See above; Comité Ético de Investigación Clinica de Galicia<br>Unidad: Subdirección Xeral de Farmacia e Produtos<br>Sanitarios. Conselleria de Sanidade

Site number	Principal investigator	IRB/IEC name, address, and committee chairperson
553	Navarro MD, Federico	See above; Comité Ético de Investigación Clinica de Galicia Unidad: Subdirección Xeral de Farmacia e Produtos Sanitarios. Conselleria de Sanidade;<br><br>See above; CEIC Autonómico de Ensayos, Clinicos de Andalucía, Conserjería de Salud;<br><br>CEIC del Hospital Universitario Virgen Macarena<br>Avenida Dr. Fedriani, 3<br>2a planta, Unidad de Investigación<br>41009 Sevilla<br>SPAIN<br>Chairperson: Dr. D. Miguel Ángel Rico Corral
554	González Gay MD, Miguel Ángel	See above: Comité Ético de Investigación Clinica de Galicia<br>Unidad: Subdirección Xeral de Farmacia e Produtos Sanitarios. Conselleria de Sanidade;<br>Comité Etico de Investigación Clinica de Cantabria<br>Fundación Marqués de Valdecilla<br>Escula Universitaria de Enfermería – planta 5a<br>Avenida de Valdecilla, s/n<br>39008 Santander (Cantabria)<br>SPAIN<br>Chairperson: D. Carlos G. Redondo Figuero

Site number	Principal investigator	IRB/IEC name, address, and committee chairperson
Central IEC<br>United Kingdom	--	Leicestershire, Northamptonshire and Rutland<br>Research Ethics Committee 2<br>The Old Chapel<br>Royal Standard Place<br>Nottingham<br>NG1 6GN<br>UNITED KINGDOM<br>Chairperson: Dr. Carl Edwards
600	Marzo-Ortega MD, Helena	See above; Leicestershire, Northamptonshire and Rutland Research Ethics Committee 2;<br><br>Leeds Teaching Hospital NHS Trust<br>Research & Development Directorate<br>34 Hyde Terrace<br>Leeds<br>LS2 9LN<br>UNITED KINGDOM<br>Chairperson: Dr. Steve Smye

Site number	Principal investigator	IRB/IEC name, address, and committee chairperson
601	Cooper MD, Robert	See above; Leicestershire, Northamptonshire and Rutland Research Ethics Committee 2;<br><br>Salford Royal NHS Foundation Trust<br>Research & Development Department<br>Summerfield House<br>554 Eccles New Road<br>Salford<br>M5 5AP<br>UNITED KINGDOM<br>Chairperson: Prof. Bill Ollier
602	Donnelly MD, Simon<br>(did not enroll)	See above; Leicestershire, Northamptonshire and Rutland Research Ethics Committee 2;<br><br>Whipps Cross University Hospital<br>Research & Development Unit<br>Room 24, Willow Lodge<br>Leytonstone<br>London<br>E11 1NR<br>UNITED KINGDOM<br>Chairperson: Mr. James Green

Site number	Principal investigator	IRB/IEC name, address, and committee chairperson
603	O'Reilly MD, David<br>(did not enroll)	See above; Leicestershire, Northamptonshire and Rutland Research Ethics Committee 2;<br><br>West Suffolk Hospital NHS Trust<br>Research & Development Office<br>Bury St. Edmonds<br>IP33 2QZ<br>UNITED KINGDOM<br>Chairperson: Dr. John Hall
604	Wilson MD, Anthony Gerard<br>(did not enroll)	See above; Leicestershire, Northamptonshire and Rutland Research Ethics Committee 2;<br><br>Sheffield Teaching Hospital NHS Trust<br>Research & Development<br>1st Floor<br>11 Brommfield Road<br>Sheffield<br>S10 2SE<br>UNITED KINGDOM<br>Chairperson: Prof. Simon Heller

Site number	Principal investigator	IRB/IEC name, address, and committee chairperson
605	Adebajo MD, Adewale	See above; Leicestershire, Northamptonshire and Rutland Research Ethics Committee 2;<br><br>Barnsley Hospital<br>Research & Development Directorate<br>Gawber Road<br>Barnsley<br>S75 2EP<br>UNITED KINGDOM<br>Chaiperson: Prof. Stuart G. Parker
Central IEC<br>Argentina	--	Comité Independiente de Ética para Ensayos en Farmacología Clínica<br>J. E. Uriburu 774, 1st Floor, Apt. C<br>(C1027AAP) Ciudad Autónoma de Buenos Aires<br>ARGENTINA<br>Chairperson: Prof. Dr. Luis M. Zieher
700	Maldonado Cocco MD, José Antonio	See above; Comité Independiente de Ética para Ensayos en Farmacología Clínica;<br><br>Comité de Docencia e Investigación de Consultorios Reumatológicos Pampa<br>La Pampa 1548, 1st Floor, Apt. A<br>(C1428DZF) Ciudad Autónoma de Buenos Aires<br>ARGENTINA<br>Chairperson: Dr. Alberto Rodriguez Velez

Site number	Principal investigator	IRB/IEC name, address, and committee chairperson
701	Eimon MD, Alicia	Comité de Ética en Investigación;<br>Centro de Educación Médica e Investigaciones Clínicas<br>"Norberto Quirno" (CEMIC)<br>Galvan 4102<br>(C1431FWO) Ciudad Autónoma de Buenos Aires<br>ARGENTINA<br>Chairperson: Prof. Enrique Gadow
702	Spindler MD, Alberto Jorge	See above; Comité Independiente de Ética para Ensayos en Farmacología Clínica;<br><br>Comité de Docencia e Investigación<br>Centro Médico Privado de Reumatología<br>Lavalle 506<br>(T4000AXL) San Miguel de Tucumán<br>Tucumán<br>ARGENTINA<br>Chairperson: Prof. Dra. Sofía Amenabar
704	Mysler MD, Eduardo F.	See above; Comité Independiente de Ética para Ensayos en Farmacología Clínica;<br><br>Comité de Docencia e Investigación<br>Organización Médica para la Investigación (OMI)<br>Uruguay 725, Ground Floor<br>(C1015ABO) Ciudad Autónoma de Buenos Aires<br>ARGENTINA<br>Chairperson: Dr. Marcelo Radisic

Site number	Principal investigator	IRB/IEC name, address, and committee chairperson
705	Alvarellos MD, Alejandro	Comité de Revision Interna<br>Hospital Privado Centro Médico de Cordoba<br>Naciones Unidas 346<br>(5016) Córdoba<br>Córdoba<br>ARGENTINA<br>Chairperson: Dr. Sergio Metrebián
706	Pons Estel MD, Bernado Antonio	See above; Comité Independiente de Ética para Ensayos en Farmacología Clínica;<br><br>Comité de Docencia e Investigación del Sanatorio Parque<br>Boulevard Oroño 860<br>(2000) Rosario<br>Sante Fe<br>ARGENTINA<br>Chairperson: Dr. Carlos Lovesio

Site number	Principal investigator	IRB/IEC name, address, and committee chairperson
707	Rillo MD, Oscar L.<br>(did not enroll)	See above; Comité Independiente de Ética para Ensayos en Farmacología Clínica;<br><br>Comité de Ética en Investigación<br>Hospital Sirio Libanes<br>Campana 4658<br>(C1419AHN) Ciudad Autónoma de Buenos Aires<br>ARGENTINA<br>Chairperson: Dr. Roberto Cataldi Amatriain;<br><br>Comité de Docencia e Investigación<br>Hospital Sirio Libanes<br>Campana 4658<br>(C1419AHN) Ciudad Autónoma de Buenos Aires<br>ARGENTINA<br>Chairperson: Dr. Gustavo Frechtel
708	Lucero MD, Eleonora	Comité de Ética Independiente;<br>Centro de Investigaciones Reumatológicas<br>Las Piedras 108<br>(T4000BRD) San Miguel de Tucumán<br>Tucumán<br>ARGENTINA<br>Chairperson: Dra. Gabriela Eva Maria Perez

Site number	Principal investigator	IRB/IEC name, address, and committee chairperson
709	Granel MD, Amelia Beatriz<br>(did not enroll)	Comité de Ética de DER Investigaciones Clínicas<br>Vicente Lopez 1441<br>(B1878DVN) Quilmes<br>Buenos Aires<br>ARGENTINA<br>Chairperson: Abog. Damián Edgardo Del Percio
710	Pardo Hidalgo MD, Rodolfo Ariel<br>(did not enroll)	See above; Comité Independiente de Ética para Ensayos en Farmacología Clínica;<br><br>Comité de Docencia e Investigación del Centro Polivalente de Asístencia e Investigación Clínica CER San Juan<br>Lapride 568 Este<br>(5400) San Juan<br>San Juan<br>ARGENTINA<br>Chairperson: Dr. Luis Angel Castro
Central IEC<br>Brazil	--	Conselho Nacional de Ética em Pesquisa – CONEP<br>Esplanada dos Ministérios<br>Bloco G, Anexo B – sala 436 b<br>Brasilia DF<br>Cep: 70.058-900<br>BRAZIL<br>Chairperson: Dr. Gyselle Saddi Tannous

Site number	Principal investigator	IRB/IEC name, address, and committee chairperson
750	Azevedo MD, Valderílio Feijó	See above; Conselho Nacional de Ética em Pesquisa – CONEP;<br><br>Comité de Ética em Pesquisa em Seres Humanos<br>Centro de Estudos Superiores Positivo – UNICENP/PR<br>Rua Pedro Viriato Parigot de Souza, 5300<br>Curítiba PR<br>Cep: 81280-330<br>BRAZIL<br>Chairperson: Dr. Maria Fernanda Torres
753	Keiserman MD, Mauro Waldemar<br>(did not enroll)	See above; Conselho Nacional de Ética em Pesquisa – CONEP;<br><br>Comité de Ética em Pesquisa da Pontifícia Universidade Católica do Rio Grande do Sul – PUC/RS<br>Av Ipiranga, 6690<br>3o andar<br>Porto Alegre RS<br>Cep: 90610-000<br>BRAZIL<br>Chairperson: Dr. Rodolfo Herberto Schneider

Site number	Principal investigator	IRB/IEC name, address, and committee chairperson
755	Zerbini MD, Cristiano Augusto de Freitas (did not enroll)	See above; Conselho Nacional de Ética em Pesquisa-CONEP;<br>UGAI – Unidade de Gestao Assistencial<br>Hospital Heliopolis<br>Comité de Ética em Pesquisa<br>Rua Conego Xavier<br>276 – 10oandar, Sacoma<br>Sao Paulo SP<br>Cep: 04231-030<br>BRAZIL<br>Chairperson: Dr. Jozias de Andrade Sobrinho
756	Chahade MD, William Habib	See above; Conselho Nacional de Ética em Pesquisa – CONEP;<br>Comité de Ética em Pesquisa do Instituto de Assistência Médica ao Servidor Público Esadual / IAMSPE / HSPE – "FMO"<br>Rua Pedro de Toledo, 1800<br>3° andar – ala central<br>Sáo Paulo SP<br>Cep:04039-901<br>BRAZIL<br>Chairperson: Dr. Roberto Dantas Queiroz

Site number	Principal investigator	IRB/IEC name, address, and committee chairperson
757	da Silva MD, Nîlzio A.<br>(did not enroll)	See above; Conselho Nacional de Ética em Pesquisa – CONEP;<br><br>Comité de Ética em Pesquisa em Seres Humanos das Clínicas da Universidade Federal de Goiás/GO<br>Primeíra Venida, s/n, Setor Leste Universitário<br>2° andar – UPC – Unidade de Pesquisa Clínica<br>Goiâna GO<br>Cep: 74605-050<br>BRAZIL<br>Chairperson: Dr. João Carlos da Rocha Medrado
760	Bertolo MD, Manoel Barros	See above; Conselho Nacional de Ética em Pesquisa – CONEP;<br><br>Comité de Ética em Pesquisa – UNICAMP<br>Rua Tessália Vieira de Camargo, 126<br>Campinas SP<br>Cep: 13083-887<br>BRAZIL<br>Chairperson: Dr. Carlos Eduardo Steiner
761	Ximenes MD, Antonio Carlos	See above; Conselho Nacional de Ética em Pesquisa – CONEP;<br><br>Comité de Ética em Pesquisa do Hospital Geral de Goiâna<br>Av. Anhanguera, 6479 – Setor Oeste<br>Goiâna GO<br>Cep: 74110-010<br>BRAZIL<br>Chairperson: Dr. Fábio Péclat do Santos

Site number	Principal investigator	IRB/IEC name, address, and committee chairperson
801	Esquivel Valerio MD, Jorge Antonio	Comité de Ética de la Facultad de Medicina de la Universidad Autónoma de Nuevo León (UANL). y Hospital Universitario "Dr. José Eleuterio González"<br>Av. Francisco I. Madero y Av. Dr. Aguirre Pequeño s/n<br>Col. Mitras Centro<br>C.P. 64460, Monterrey, Nuevo León<br>MEXICO<br>Chairperson: Dr. José Gerardo Garza Leal
802	Xibillé Friedmann MD, Daniel Xavier	Comité de Ética en Investigación del Hospital Inovamed<br>Calle Cuauhtémoc #305, Col. Lomas de la Selva<br>C.P. 62270, Cuernavaca Morelos<br>MEXICO<br>Chairperson: Dr. Bernardo J. Rubio Cano
803	García García MD, Conrado<br>(did not enroll)	Comité Bioético para la Investigación Clinica, S.C.<br>Puebla # 422-4, Col. Roma Sur, Del . Cuahtémoc<br>C.P. 06700, México, D.F.<br>MÉXICO<br>Chairperson: Biol. Celia Ovadia Savariego
Central IEC<br>Canada	--	Quorum Review Insitutional Review Board<br>1601 Fifth Avenue, Suite 100<br>Seattle, WA 98101<br>UNITED STATES<br>Chairperson: David B. Kelley, MD
900	Khraishi MD, Majed Mustafa	See above; Quorum Review Institutional Review Board

Site number	Principal investigator	IRB/IEC name, address, and committee chairperson
901	Rahman MD, Al-Amin Proton<br>(did not enroll)	Human Investigation Committee<br>2nd Floor, Eastern Trust Building<br>95 Bonavenutre Avenue<br>St. John's, NL A1B 2X5<br>CANADA<br>Chairpersons: John Harnett, MD (co-chair) and Fern Brunger, MD (co-chair)
902	Beaulieu MD, André<br>(did not enroll)	See above; Quorum Review Institutional Review Board
903	Thomson MD, Glen T. D.	See above; Quorum Review Institutional Review Board
905	Tremblay MD, Jean-Luc<br>(did not enroll)	See above; Quorum Review Institutional Review Board
906	Inman MD, Robert Davies<br>(did not enroll)	University Health Network Research Ethics Board<br>Hydro Building, 10th Floor, Suite 1056<br>700 University Avenue<br>Toronto, ON M5G 1Z5<br>CANADA<br>Chairperson: Karen McRae, MD
907	Baker MD, Milton F.	See above; Quorum Review Institutional Review Board
908	Keystone MD, Edward C.<br>(did not enroll)	Mount Sinai Hospital Research Ethics Board<br>600 University Avenue<br>Toronto, ON M5X 1G5<br>CANADA<br>Chairperson: Ron Heslegrave, MD
910	Rodrigues MD, Jude F.	See above; Quorum Review Institutional Review Board

Site number	Principal investigator	IRB/IEC name, address, and committee chairperson
912	Haraoui MD, Boulos<br>(did not enroll)	See above; Quorum Review Institutional Review Board
Central IEC<br>United States	--	Quorum Review Institutional Review Board<br>1601 Fifth Avenue, Suite 100<br>Seattle, WA 98101<br>UNITED STATES<br>Chairperson: David B. Kelley, MD
950	Baldassare MD, Andrew<br>(replaced by Akgun Ince, MD)<br>Ince MD, Akgun<br>(replaced Andrew Baldassare, MD)	See above; Quorum Review Institutional Review Board
951	Diab MD, Isam A.	See above; Quorum Review Institutional Review Board
952	Dikranian MD, Ara<br>(did not enroll)	See above; Quorum Review Institutional Review Board
953	Jones MD, Richard E., III<br>(did not enroll)	See above; Quorum Review Institutional Review Board
954	Schechtman DO, Joy	See above; Quorum Review Institutional Review Board
956	Bagheri MD, Shahin<br>(did not enroll)	See above; Quorum Review Institutional Review Board
957	Gaylis MD, Norman B.	See above; Quorum Review Institutional Review Board
958	Kennedy MD, Alastair C.	See above; Quorum Review Institutional Review Board
959	Oza MD, Meera R.	See above; Quorum Review Institutional Review Board
960	Swartz MD, Timothy J.<br>(did not enroll)	See above; Quorum Review Institutional Review Board
961	Turkiewicz MD, Anthony M.	See above; Quorum Review Institutional Review Board

Site number	Principal investigator	IRB/IEC name, address, and committee chairperson
962	Sherrer MD, Yvonne R.	See above; Quorum Review Institutional Review Board
963	Gough MD, William	See above; Quorum Review Institutional Review Board
964	Howell MD, Mary P.	See above; Quorum Review Institutional Review Board
965	Rosenberg MD, David S.<br>(did not enroll)	See above; Quorum Review Institutional Review Board
966	Greenwald MD, Maria W.	See above; Quorum Review Institutional Review Board
967	Stolow MD, Joshua B.	See above; Quorum Review Institutional Review Board
968	Mease MD, Philip J.	Western Institutional Review Board<br>3535 Seventh Avenue SW<br>Olympia, WA 98502<br>UNITED STATES<br>Chairperson: Theodore D. Schultz, JD
969	Moreta Freire MD, Elvia G.	See above; Quorum Review Institutional Review Board
970	Codding MD, Christine E.	See above; Quorum Review Institutional Review Board
971	Rizzo MD, Warren C.<br>(did not enroll)	See above; Quorum Review Institutional Review Board
972	Kivitz MD, Alan J.	See above; Quorum Review Institutional Review Board
973	Wallace MD, Daniel J.<br>(did not enroll)	Cedars Sinai Medical Center Institutional Review Board<br>8383 Wilshire Boulevard, Suite 742<br>Beverly Hills, CA 90211<br>UNITED STATES<br>Chairperson: Stephen Lim, MD

Site number	Principal investigator	IRB/IEC name, address, and committee chairperson
974	Kavanaugh MD, Arthur F.	University of California, San Diego Human Research Protection Program<br>8950 Villa La Jolla Drive, Suite A 208<br>La Jolla, CA 92037<br>UNITED STATES<br>Chairperson: Michael Caligiuri, PhD
975	Fleischmann MD, Roy M.	See above; Quorum Review Institutional Review Board
977	Khan MD, Muhammed Asim<br>(did not enroll)	MetroHealth Medical Center Institutional Review Board<br>2500 MetroHealth Drive, Room 103 Rammelkamp<br>Cleveland, OH 44109<br>UNITED STATES<br>Chairperson: David Kuentz, DO, MBA
978	Wellborne DO, Frank R.<br>(did not enroll)	See above; Quorum Review Institutional Review Board
981	Walsh MD, Jessica	University of Utah Institutional Review Board<br>Research Administration Building<br>75 South 2000 East<br>Salt Lake City, UT 84112<br>UNITED STATES<br>Chairperson: John Stillman
982	Deodhar MD, Atulya A.	Oregon Health & Science University Institutional Review Board<br>Mail Code L1 06, 3181 SW Sam Jackson Park Road<br>Portland, OR 97239<br>UNITED STATES<br>Chairperson: Susan B. Bankowski, MS, JD

Site number	Principal investigator	IRB/IEC name, address, and committee chairperson
983	Reveille MD, John D.	Committee for the Protection of Human Subjects<br>6410 Fannin Street, Suite 1100<br>Houston, TX 77030<br>UNITED STATES<br>Chairperson: George Stancel, PhD
984	Hensarling MD, James Kenneth<br>(did not enroll)	See above; Quorum Review Institutional Review Board
985	Goddard MD, David Harry	See above; Quorum Review Institutional Review Board
986	Kremer MD, Joel M.<br>(did not enroll)	See above; Quorum Review Institutional Review Board
987	Maricic MD, Michael	See above; Quorum Review Institutional Review Board
989	Schnitz MD, William M.<br>(did not enroll)	See above; Quorum Review Institutional Review Board
990	Figueroa MD, Debra P.	See above; Quorum Review Institutional Review Board
