# Supplementary material for: Discriminant validity, responsiveness and reliability of the arthritis-specific Work Productivity Survey assessing workplace and household productivity within and outside the home in patients with axial spondyloarthritis, including nonradiographic axial spondyloarthritis and ankylosing spondylitis
Source: Arthritis Res Ther. 2014 Aug 6;16(4):R164. doi: 10.1186/ar4680 (PMC4448884; doi:10.1186/ar4680)
Supplement: Supplementary file 2 — Additional file 2: Questionnaires assessed. Details of the questionnaires assessed in the article. (DOCX 29 KB) [file 13075_2013_4379_MOESM2_ESM.docx]

| **Questionnaire** | **Description** | **References** |
| --- | --- | --- |
| ASDAS | The ASDAS is a composite score, derived from a number of assessments which are scored by the subject and physician and multiplied by a proven formula. The ASDAS score is generated by a weighted sum of the components (back pain [BASDAI question 2], Patient’s Global Assessment of Disease Activity [PtGADA], duration of morning stiffness [BASDAI question 6], peripheral pain/swelling [BASDAI question 3], fatigue and C-reactive protein [CRP]), with lower scores indicating low disease activity. | [[1](#_ENREF_1)] |
| BASFI | The BASFI comprises 10 items assessing physical function over the preceding week. The first 8 questions evaluate activities related to functional anatomical limitations due to the course of this inflammatory disease. The final 2 questions evaluate the patients’ ability to cope with everyday life. Each item is rated on a 0 to 10 numeric rating scale (NRS), where 0=easy and 10=impossible. The summary score from this scale is the mean of the 10 items and ranges from 0 to 10, with 0 representing the best state (lower disease activity) and 10 the worst state. The minimum clinically important difference (MCID) for BASFI is 1 point. | [[2-4](#_ENREF_2)] |
| BASDAI | The BASDAI is the most commonly used instrument to measure the disease activity of ankylosing spondylitis over the preceding week. The BASDAI is a validated, self-reported instrument which consists of 6 horizontal NRS, each with 10 units to measure the severity of the 5 major symptoms: fatigue, spinal pain, peripheral joint pain and swelling, enthesitis, and morning stiffness (both severity and duration, respectively) over the last week. Items 1 to 5 are rated on a 0 to 10 NRS, where 0=none and 10=very severe. Item six is rated from 0=0 hr to 10=2 or more hours. The summary score is a weighted mean of the six items, and ranges from 0 to 10, with 0 representing the best state and 10 the worst state.  The BASDAI50 is defined as an improvement of at least 50% in the BASDAI compared to baseline. A response criterion for the BASDAI is defined by an MCID decrease of at least 1. | [[4](#_ENREF_4)] |
| Total and Nocturnal Spinal Pain | Total and Nocturnal Spinal Pain are assessed by two questions rated on a 0 to 10 NRS, where 0=no pain and 10=most severe pain. Total spine pain is assessed by ‘How much pain in your spine due to spondyloarthritis do you have?’, and nocturnal spine pain: ‘How much pain in your spine due to spondyloarthritis do you have at night? | - |
| ASQoL | The ASQoL is an 18-item questionnaire, each item assessing the patient’s current opinion. Each item is scored as 1=yes or 0=no. The summary score is the total of the yes/no scores, thus ranging from 0 (best HRQoL) to 18 (worst HRQoL). | [[5](#_ENREF_5)] |
| SF-36 | The SF-36 is a widely used generic HRQoL instrument that evaluates eight health domains: physical functioning, role physical, bodily pain, general health, vitality, social functioning, role emotional, and mental health. The eight domains are summarized in two component summaries: the Physical Component Summary (PCS) and the Mental Component Summary (MCS). Scores for the SF-36 range between 0 and 100, with higher scores indicating a better HRQoL. | [[6](#_ENREF_6)] |
| EQ-5D | The EQ-5D questionnaire is comprised of a 5-item health status measure and a VAS scale. Each of the 5 dimensions is divided into 3 levels: no problem, some or moderate problems, and extreme problems and is scored as 1, 2, and 3, respectively. The EQ-5D VAS records the respondent’s self-rated health status on a vertical 20 cm scale, graduated from 0 to 100 (0=worst imaginable health status, 100=best imaginable health status). | [[7](#_ENREF_7), [8](#_ENREF_8)] |
| ASAS20/40/50/70 | The ASAS20 response is defined as an improvement of at least 20% and absolute improvement of at least 1 unit on a 0 to 10 NRS in at least 3 of 4 domains: PtGADA, the total spinal pain NRS score, BASFI, and the mean of BASDAI questions 5 and 6 concerning morning stiffness intensity and duration and the absence of deterioration in the potential remaining domain [deterioration is defined as a relative worsening of at least 20% and an absolute worsening of at least 1 unit].  The ASAS criteria for 40%, 50%, or 70% improvement are defined as relative improvements of at least 40%, 50%, or 70% and absolute improvement of at least 2 units, on a 0 to 10 NRS, in at least 3 of the 4 domains and no worsening at all in the remaining domain. | [[9](#_ENREF_9)] |

**References**

1. van der Heijde D, Lie E, Kvien TK, Sieper J, Van den Bosch F, Listing J, Braun J, Landewe R: **ASDAS, a highly discriminatory ASAS-endorsed disease activity score in patients with ankylosing spondylitis**. *Annals of the rheumatic diseases* 2009, **68**(12):1811-1818.

2. Calin A, Garrett S, Whitelock H, Kennedy LG, O'Hea J, Mallorie P, Jenkinson T: **A new approach to defining functional ability in ankylosing spondylitis: the development of the Bath Ankylosing Spondylitis Functional Index**. *The Journal of rheumatology* 1994, **21**(12):2281-2285.

3. van Tubergen A, Debats I, Ryser L, Londoño J, Burgos-Vargas R, Cardiel MH, Landewé R, Stucki G, Van der Heijde D: **Use of a numerical rating scale as an answer modality in ankylosing spondylitis–specific questionnaires**. *Arthritis Care & Research* 2002, **47**(3):242-248.

4. Pavy S, Brophy S, Calin A: **Establishment of the minimum clinically important difference for the bath ankylosing spondylitis indices: a prospective study**. *The Journal of rheumatology* 2005, **32**(1):80-85.

5. Doward LC, Spoorenberg A, Cook SA, Whalley D, Helliwell PS, Kay LJ, McKenna SP, Tennant A, van der Heijde D, Chamberlain MA: **Development of the ASQoL: a quality of life instrument specific to ankylosing spondylitis**. *Annals of the rheumatic diseases* 2003, **62**(1):20-26.

6. Ware JE, Jr., Sherbourne CD: **The MOS 36-item short-form health survey (SF-36). I. Conceptual framework and item selection**. *Medical care* 1992, **30**(6):473-483.

7. Brooks R: **EuroQol: the current state of play**. *Health policy* 1996, **37**(1):53-72.

8. EuroQol G: **EuroQol--a new facility for the measurement of health-related quality of life**. *Health policy* 1990, **16**(3):199-208.

9. Anderson JJ, Baron G, Van Der Heijde D, Felson DT, Dougados M: **Ankylosing spondylitis assessment group preliminary definition of short-term improvement in ankylosing spondylitis**. *Arthritis & Rheumatism* 2001, **44**(8):1876-1886.
